# Association of intraoperative end-tidal CO_2_ levels with postoperative outcomes: a patient-level analysis of two randomised clinical trials

**DOI:** 10.1016/j.bja.2025.07.076

**Published:** 2025-08-14

**Authors:** Prashant Nasa, David M.P. van Meenen, Frederique Paulus, Carlos Ferrando, Thomas Bluth, Marcelo Gama de Abreu, Lorenzo Ball, Sebastiaan M. Bossers, Patrick Schober, Marcus J. Schultz, Ary Serpa Neto, Sabrine N.T. Hemmes, for the REPEAT, Niklas S. Campos, Thomas Bluth, Sabrine N.T. Hemmes, Julian Librero, Natividad Pozo, Carlos Ferrando, Lorenzo Ball, Guido Mazzinari, Paolo Pelosi, Marcelo Gama de Abreu, Marcus J. Schultz, Ary Serpa Neto, the PROVHILO, Sabrine N.T. Hemmes, Marcelo Gama de Abreu, Paolo Severgnini, Markus W. Hollmann, Jan M. Binnekade, Hermann Wrigge, Jaume Canet, Michael Hiesmayr, Werner Schmid, Edda Tschernko, Samir Jaber, Göran Hedenstierna, Christian Putensen, Paolo Pelosi, Marcus J. Schultz, Agnes Marti, Alessandro Bacuzzi, Alexander Brodhun, Alexandre Molin, Alfred Merten, Ana Parera, Andrea Brunelli, Andrea Cortegiani, Andreas Güldner, Andreas W. Reske, Angelo Gratarola, Antonino Giarratano, Bea Bastin, Bjorn Heyse, Branka Mazul-Sunko, Bruno Amantea, Bruno Barberis, Christian Putensen, Christopher Uhlig, Conrado Minguez Marín, Cristian Celentano, Daniela La Bella, David D’Antini, David Velghe, Demet Sulemanji, Edoardo De Robertis, Eric Hartmann, Francesca Montalto, Francesco Tropea, Gary H. Mills, Gilda Cinnella, Giorgio Della Rocca, Girolamo Caggianelli, Giulia Pellerano, Giuseppina Mollica, Guillermo Bugedo, Hermann Wrigge, Jan-Paul Mulier, Jeroen Vandenbrande, Johann Geib, Jonathan Yaqub, Jorge Florez, Juan F. Mayoral, Juraj Sprung, Jurgen Van Limmen, Lieuwe D.J. Bos, Luc de Baerdemaeker, Luc Jamaer, Luigi Spagnolo, Lydia Strys, Manuel Granell Gil, Marcelo Gama de Abreu, Marcos F. Vidal Melo, Marcus J. Schultz, Maria Carmen Unzueta, Maria Victoria Moral, Marion Ferner, Markus W. Hollmann, Martin Weiss, Massimo Vanoni, Maximilian S. Schaefer, Mercè Prieto, Michele Grio, Paolo Severgnini, Peter Markus Spieth, Philipp Simon, Phoebe Bodger, Pilar Sierra, Rita Laufenberg-Feldmann, Roberta Rusca, Rodolfo Proietti, Sabrine N.T. Hemmes, Santi Maurizio Raineri, Santo Caroleo, Sergi Sabaté, Stefan De Hert, Stefano Pezzato, Tanja A. Treschan, Tatjana Goranovic, Thea Koch, Thomas Bluth, Thomas Kiss, Valter Perilli, Virginia Cegarra, Werner Schmid, the PROBESE, Thomas Bluth, Ary Serpa Neto, Ilona Bobek, Jaume Canet, Gilda Cinnella, Luc de Baerdemaeker, Cesare Gregoretti, Göran Hedenstierna, Sabrine N.T. Hemmes, Michael Hiesmayr, Markus W. Hollmann, Samir Jaber, John Laffey, Marc-Joseph Licker, Klaus Markstaller, Idit Matot, Gary H. Mills, Jan Paul Mulier, Christian Putensen, Rolf Rossaint, Jochen Schmitt, Mert Senturk, Paolo Severgnini, Juraj Sprung, Marcos F. Vidal Melo, Hermann Wrigge, Marcus J. Schultz, Paolo Pelosi, Marcelo Gama de Abreu, Fernando Abelha, Sühayla Abitağaoğlu, Marc Achilles, Afeez Adebesin, Ine Adriaensens, Charles Ahene. Fatima Akbar, Mohammed Al Harbi, Rita Al Khoury al Kallab, Xavier Albanel, Florence Aldenkortt, Rawan Abdullah Saleh Alfouzan, Reef Alruqaie, Fernando Altermatt, Bruno Luís de Castro Araujo, Arbesú Genaro, Hanna Artsi, Caterina Aurilio. Omer Hilmi Ayanoglu, Alessandro Bacuzzi, Harris Baig, Yolanda Baird, Konstantin Balonov. Jaume Balust, Samantha Banks, Xiaodong Bao, Mélanie Baumgartner, Isabel Belda Tortosa, Alice Bergamaschi, Lars Bergmann, Luca Bigatello, Elena Biosca Pérez, Katja Birr, Thomas Bluth, Elird Bojaxhi, Chiara Bonenti, Iwona Bonney, Elke M.E. Bos, Sara Bowman, Leandro Gobbo Braz, Elisa Brugnoni, Sorin J. Brull, Iole Brunetti, Andrea Bruni, Shonie L. Buenvenida, Cornelius Johannes Busch, Giovanni Camerini, Jaume Canet, Beatrice Capatti, Javiera Carmona, Jaime Carungcong, Marta Carvalho, Anat Cattan, Carla Cavaleiro, Davide Chiumello, Stefano Ciardo, Mark Coburn, Umberto Colella, Victor Contreras, Pelin Corman Dincer, Elizabeth Cotter, Marcia Crovetto, William Darrah, Simon Davies, Luc de Baerdemaeker, Stefan De Hert, Enrique Del Cojo Peces, Ellise Delphin, John Diaper, Paulo do Nascimento Junior, Valerio Donatiello, Jing Dong, Maria do Socorro Dourado, Alexander Dullenkopf, Felix Ebner, Hamed Elgendy, Christoph Ellenberger, Dilek Erdoğan Arı, Thomas Ermert, Fadi Farah, Ana Fernandez-Bustamante, Cristina Ferreira, Marco Fiore, Ana Fonte, Christina Fortià Palahí, Andrea Galimberti, Marcelo Gama de Abreu, Najia Garofano, Luca Gregorio Giaccari, Fernando Gilsanz, Felix Girrbach, Luca Gobbi, Marc Bernard Godfried, Nicolai Goettel, Peter A. Goldstein, Or Goren, Andrew Gorlin, Granell Gil Manuel, Angelo Gratarola, Juan Graterol, Pierre Guyon, Kevin Haire, Philippe Harou, Antonia Helf, Sabrine N.T. Hemmes, Gunther Hempel, María José Hernández Cádiz, Björn Heyse, Markus W. Hollmann, Ivan Huercio, Jasmina Ilievska, Lien Jakus, Vijay Jeganath, Yvonne Jelting, Minoa Jung, Barbara Kabon, Aalok Kacha, Maja Karaman Ilić, Arunthevaraja Karuppiah, Ayse Duygu Kavas, Gleicy Keli Barcelos, Todd A. Kellogg, Johann Kemper, Romain Kerbrat, Suraya Khodr, Peter Kienbaum, Bunyamin Kir, Thomas Kiss, Selin Kivrak, Vlasta Klarić, Thea Koch, Ceren Köksal, Ana Kowark, Peter Kranke, Bahar Kuvaki, Biljana Kuzmanovska, John Laffey, Mirko Lange, Marília Freitas de Lemos, Marc-Joseph Licker, Manuel López-Baamonde, Antonio López-Hernández, Mercedes Lopez-Martinez, Stéphane Luise, Mark MacGregor, Danielle Magalhães, Julien Maillard, Patrizia Malerbi, Natesan Manimekalai, Michael Margarson, Klaus Markstaller, Archer K. Martin, David P. Martin, Yvette N. Martin, Julia Martínez-Ocon, Ignacio Martin-Loeches, Emilio Maseda, Idit Matot, Niamh McAuliffe, Travis J. McKenzie, Paulina Medina, Melanie Meersch, Angelika Menzen. Els Mertens. Bernd Meurer, Tanja Meyer-Treschan. Changhong Miao. Camilla Micalizzi, Morena Milić, Norma Sueli Pinheiro Módolo, Pierre Moine, Patrick Mölders, Ana Montero-Feijoo, Enrique Moret, Markus K. Muller, Zoe Murphy, Pramod Nalwaya, Filip Naumovski. Navalesi Paolo, Lais Helena Navarro e Lima, Višnja Nesek Adam, Claudia Neumann, Christopher Newell, Zoulfira Nisnevitch, Junaid Nizamuddin, Cecilia Novazzi, Michael O'Connor, Günther Oprea, Mukadder Orhan Sungur, Şule Özbilgin, Maria Caterina Pace, Marcos Pacheco, Balaji Packianathaswamy, Estefania Palma Gonzalez, Fotios Papaspyros. Sebastián Paredes, Maria Beatrice Passavanti, Juan Cristobal Pedemonte, Paolo Pelosi. Sanja Peremin, Christoph Philipsenburg, Daniela Pinho, Silvia Pinho, Linda M. Posthuma, Vincenzo Pota, Benedikt Preckel, Paolo Priani, Christian Putensen, Mohamed Aymen Rached, Aleksandar Radoeshki, Riccardo Ragazzi, Tamilselvan Rajamanickam, Arthi Rajamohan, Harish Ramakrishna. Desikan Rangarajan. Christian Reiterer, J.Ross Renew, Thomas Reynaud, Rhidian Rhys, Eva Rivas. Luisa Robitzky, Rolf Rossaint. Francesca Rubulotta. S. Machado Humberto, S.Nunes Catarina, Giovanni Sabbatini, Jon D. Samuels, Josep Martí Sanahuja, Pasquale Sansone, Alice Santos, Mohamed Sayedalahl, Maximilian S. Schaefer, Martin Scharffenberg, Eduardom Schiffer, Nadja Schliewe, Raoul Schorer, Marcus J. Schultz, Roman Schumann, Gabriele Selmo, Mar Sendra, Mert Senturk, Paolo Severgnini. Kate Shaw, Mirjana Shosholcheva. Abdulrazak Sibai. Philipp Simon, Francesca Simonassi, Claudia Sinno, Nukhet Sivrikoz, Vasiliki Skandalou. Neil Smith, Maria Soares, Tania Socorro Artiles, Diogo Sousa Castro. Miguel Sousa, Savino Spadaro. Juraj Sprung, Emmanouil Stamatakis, Luzius A. Steiner, Andrea Stevenazzi, Alejandro Suarez-de-la-Rica. Mélanie Suppan, Robert Teichmann, José Maria Tena Guerrero. Bram Thiel, Raquel Tolós, Gulbin Tore Altun, Michelle Tucci, Zachary A. Turnbull, Žana Turudić, Matthias Unterberg, Jurgen Van Limmen, Yves Van Nieuwenhove, Julia Van Waesberghe, Marcos Francisco Vidal Melo. Bibiana Vitković, Luigi Vivona, Marcela Vizcaychipi. Carlo Alberto Volta, Anne Weber, Toby N. Weingarten, Jakob Wittenstein, Hermann Wrigge, Piet Wyffels, Julio Yagüe, David Yates, Ayşen Yavru, Lilach Zac, Jing Zhong, investigators of the PROVE network, Carlos Ferrando, Javier Belda, Marina Soro, Jaume Canet, Carmen Unzueta, Fernando Suarez-Sipmann, Julián Librero, Alicia Llombart, Lucas Rovira, Manuel Granell, César Aldecoa, Oscar Diaz-Cambronero, Jaume Balust, Ignacio Garutti, Rafael Gonzalez, Lucia Gallego, Santiago Garcia del Valle, Javier Redondo, David Pestaña, Aurelio Rodríguez, Javier García, Manuel de la Matta, Maite Ibáñez, Francisco Barrios, Samuel Hernández, Vicente Torres, Salvador Peiró, Natividad Pozo, Abigail Villena, Albert Carramiñana, Alberto Gallego-Casilda, Alejandro Duca, Amalia Alcón, Amanda Miñana, Ana Asensio, Ana Colás, Ana Isabel Galve, Ana Izquierdo, Ana Jurado, Ana María Pérez, Ana Mugarra, Ana Parera, Andrea Brunelli, Andrea Gutierrez, Ángeles De Miguel, Angels Lozano, Antonio Katime, Antonio Romero, Beatriz Garrigues, Begoña Ayas, Blanca Arocas, Carlos Delgado, Carmen Fernández, Carolina Romero, Clara Gallego, Cristina Garcés, Cristina Lisbona, Cristina Parrilla, Daniel López-Herrera, Domingo González, Eduardo Llamazares, Elena Del Rio, Elena Lozano, Ernesto Pastor, Estefanía Chamorro, Estefanía Gracia, Ester Sánchez, Esther Romero, Fernando Díez, Ferran Serralta, Francisco Daviu, Francisco Sandín, Gerardo Aguilar, Gerardo Tusman, Gonzalo Azparren, Graciela Martínez-Pallí, Guido Mazzinari, Inmaculada Benítez, Inmaculada Hernandéz, Inmaculada India, Irene León, Isabel Fuentes, Isabel Ruiz, Jaume Puig, Javie Ignacio Román, Jesús Acosta, Jesús Rico-Feijoo, Jonathan Olmedo, Jose A. Carbonell, Jose M. Alonso, Jose María Pérez, Jose Miguel Marcos, Jose Navarro, Jose Valdivia, Juan Carrizo, Laura Piqueras, Laura Soriano, Laura Vaquero, Lisset Miguel, Lorena Muñoz, Lucia Valencia, Luis Olmedilla, Ma Justina Etulain, Manuel Tisner, María Barrio, María Dolores Alonso, María García, María J. Hernández, María José Alberola, María Parra, María Pilar Argente, María Vila, Mario De Fez, Marta Agilaga, Marta Gine, Mercedes Ayuso, Mercedes García, Natalia Bejarano, Natalia Peña, Nazario Ojeda, Nilda Martínez, Nuria García, Oto Padrón, Pablo García, Paola Valls, Patricia Cruz, Patricia Piñeiro, Pedro Charco, Rafael Anaya, Ramiro López, Rayco Rodríguez, Rocío Martínez, Roger Pujol, Rosa Dosdá, Rosa Lardies, Ruben Díaz, Rubén Villazala, Sara Zapatero, Sergio Cabrera, Sergio Sánchez, Silvia Martin, Suzana Diaz, Tania Franco, Tania Moreno, Tania Socorro, Vicente Gilabert, Victor Balandrón, Victoria Moral, Virgina Cegarra, Viviana Varón, the ESAIC CTN

**Affiliations:** 1 Department of Anaesthesia and Critical Care Medicine, New Cross Hospital, The Royal Wolverhampton NHS Trust, Wolverhampton, UK; 2 Department of Intensive Care, Amsterdam University Medical Centers, location ‘AMC’, Amsterdam, The Netherlands; 3 Department of Anaesthesiology, Amsterdam University Medical Centers, location ‘AMC’, Amsterdam, The Netherlands; 4 Department of Intensive Care & Laboratory of Experimental Intensive Care and Anaesthesiology (L·E·I·C·A), Amsterdam University Medical Centers, location ‘AMC’, Amsterdam, The Netherlands; 5 Department of Anaesthesiology and Critical Care, Hospital Clinic de Barcelona, Barcelona, Spain; 6 CIBER of Respiratory Disease, Carlos III Health Institute, Madrid, Spain; 7 Pulmonary Engineering Group, Department of Anaesthesiology and Intensive Care Medicine, University Hospital Carl Gustav Carus, Technical University Dresden, Dresden, Germany; 8 Departments of Intensive Care and Resuscitation and Outcomes Research Department of Cardiothoracic Anaesthesiology, Cleveland Clinic, Cleveland, OH, USA; 9 Department of Surgical Sciences and Integrated Diagnostics (DISC), IRCCS San Martino Policlinico Hospital, University of Genoa, Genoa, Italy; 10 Department of Anaesthesiology, Amsterdam University Medical Centers, location ‘VUmc’, Amsterdam, The Netherlands; 11 Helicopter Emergency Medical Service Lifeliner 1, Amsterdam, The Netherlands; 12 Mahidol-Oxford Tropical Medicine Research Unit (MORU), Mahidol University, Bangkok, Thailand; 13 Nuffield Department of Medicine, University of Oxford, Oxford, UK; 14 Department of Anaesthesia, General Intensive Care and Pain Management, Division of Cardiothoracic and Vascular Anaesthesia & Critical Care Medicine, Medical University of Vienna, Vienna, Austria; 15 Australian and New Zealand Intensive Care Research Centre (ANZIC-RC), School of Public Health and Preventive Medicine, Monash University, Melbourne, Australia; 16 Department of Intensive Care, Melbourne Medical School, University of Melbourne, Austin Hospital, Melbourne, Australia; 17 Department of Critical Care, Melbourne Medical School, University of Melbourne, Austin Hospital, Melbourne, Australia; 18 Department of Critical Care Medicine, Hospital Israelita Albert Einstein, São Paulo, Brazil; 19 Department of Anaesthesiology, The Netherlands Cancer Institute–Antoni van Leeuwenhoek Hospital, Amsterdam, The Netherlands

**Keywords:** anaesthesia, carbon dioxide, end-tidal CO_2_, etCO_2_, intraoperative ventilation, invasive ventilation, postoperative pulmonary complications, PPCs

## Abstract

**Background:**

The relationship between intraoperative end-tidal CO_2_ (etCO_2_) levels and postoperative outcomes remains unclear. We conducted a *post hoc* analysis of two randomised trials in adults undergoing major surgery under general anaesthesia.

**Methods:**

We re-analysed individual participant data comparing high or low positive end-expiratory pressure with low tidal volume intraoperative ventilation using a merged database derived from two randomised trials in non-obese (PROVHILO: ISRCTN70332574) and obese (PROBESE: NCT02148692) patients. The exposure of interest was low etCO_2_ (<4.7 kPa) *vs* normal-high etCO_2_ (≥4.7 kPa). The primary outcome was postoperative pulmonary complications within 5 days. A time-weighted etCO_2_ analysis and propensity score matching were also performed to adjust for confounding.

**Results:**

Of 2793 participants, 891 (29.4%; 52% female) had low etCO_2_, compared with 1972/2793 (70.6%; 65% female) participants with normal-high etCO_2_. Compared with participants with normal-high etCO_2_, higher minute volumes (normalised to body weight) were delivered in participants with low etCO_2_. Postoperative pulmonary complications developed in 278/821 (34%) participants with low etCO_2_, compared with 462/1972 (23%) participants who had normal-high etCO_2_ (adjusted hazard ratio, 1.3; 95% confidence interval, 1.1–1.6; *P*<0.001). The time-weighted analysis showed an inverse linear relationship between the mean etCO_2_ and postoperative pulmonary complications, which was also confirmed by propensity matching.

**Conclusions:**

Low etCO_2_ occurs often during intraoperative ventilation and is associated with a higher rate of PPCs. The etCO_2_ level has an inverse dose-dependent relationship with postoperative pulmonary complications.

**Clinical trial registration:**

NCT05550181.

**Editor’s key points**

• The relationship between intraoperative end-tidal CO_2_ (etCO_2_) levels and postoperative outcomes remains unclear.
• The authors hypothesised that time-weighted, low etCO_2_ (<4.7 kPa) was linked to postoperative pulmonary complications after major surgery under general anaesthesia.
• The authors conducted an individual participant analysis of two randomised trials.
• Low etCO_2_ was associated with ∼50% more postoperative pulmonary complications.
• These data require prospective randomised validation to establish whether etCO_2_ 4.7-6.0 kPa should be a ventilatory management target.

Patients receiving intraoperative ventilation during general anaesthesia for surgery often have low end-tidal CO_2_ (etCO_2_) levels.^1^, ^2^, ^3^, ^4^, ^5^, ^6^, ^7^ This suggests that anaesthesiologists use excessively high tidal volume (V_T_), excessively high respiratory rates (RRs), or both.^1^, ^2^, ^3^ Lower intraoperative etCO_2_ levels are associated with postoperative mortality^5^ and hospital length of stay.^5^, ^6^, ^7^

However, the relationships between intraoperative etCO_2_ levels and patient factors, types of surgery, and ventilation characteristics remain uncertain. It is also unclear whether the association between lower etCO_2_ levels (<4.7 kPa) and poorer postoperative outcomes is independent of these factors. Therefore, we conducted an individual patient-level analysis of two randomised clinical studies of intraoperative ventilation, in which high positive end-expiratory pressure (PEEP) was compared with low PEEP at a low V_T_, in non-obese^8^ and obese^9^ patients.

The specific aims of this *post hoc* analysis were two-fold. First, we assessed the incidence of intraoperative low etCO_2_ levels and its relation to patient characteristics, types of surgery, and intraoperative ventilation management. Second, we determined the association between intraoperative low etCO_2_ levels and the development of postoperative pulmonary complications. We hypothesised that intraoperative low etCO_2_ levels depend on patient characteristics, types of surgery, and intraoperative ventilation management, and that intraoperative low etCO_2_ levels have an independent association with the development of PPCs.

## Methods

### Study design

The protocol and statistical analysis plan for this study was published at clinicaltrials.gov (NCT05550181). This is a *post hoc* analysis of a merged database named ‘Re-evaluation of the Effects of High PEEP with Recruitment Manoeuvres versus Low PEEP without Recruitment Manoeuvres During General Anaesthesia for Surgery’ (REPEAT; NCT03937375).^10^ The REPEAT database contains intraoperative and postoperative data of individual patients randomised in the ‘High versus Low Positive End-expiratory Pressure During General Anaesthesia for Open Abdominal Surgery study’ (PROVHILO)^8^ and the ‘PRotective Ventilation with Higher versus Lower PEEP during General Anaesthesia for Surgery in OBESE Patients study’ (PROBESE).^9^ This database also contains data from patients randomised in the ‘Individualized perioperative open lung ventilatory strategy study’ (iPROVE),^11^ but these patients were not included in the current analysis, as this study did not capture etCO_2_.

The study protocol of PROVHILO was approved by the institutional review board of the Amsterdam University Medical Centers, location AMC, Amsterdam, The Netherlands (reference number: METC 10/251311.17.417; ISRCTN70332574). The study protocol of PROBESE was approved by the institutional review board of the Technical University Dresden, Dresden, Germany (reference number: BO-EK 515102021) and registered at ClinicalTrials.gov (identifier NCT02148692). In both studies, written informed consent was obtained from all study subjects. Both studies adhered to the principles of Good Clinical Practice.

### Inclusion criteria

Participants were eligible for participation in PROVHILO or PROBESE if they were (1) aged >18 yr; (2) at an intermediate to high risk for PPCs according to the ‘Assess Respiratory Risk in Surgical Patients in Catalonia for Postoperative Pulmonary Complications’ (ARISCAT) risk score (≥26); and (3) planned for major surgery with an expected duration ≥2 h.

### Exclusion criteria

The PROVHILO excluded patients with a BMI >40 kg m^−2^ and PROBESE excluded patients with a BMI <35 kg m^−2^. Additional exclusion criteria are detailed in Supplementary Table S1. For the current analysis, we also excluded patients who underwent urgent or emergency surgery, because we considered it likely that these patients may have had metabolic abnormalities at the time of surgery for which anaesthesiologists may have adjusted intraoperative ventilator management. We also excluded patients with missing etCO_2_ recordings.

### Data collection and calculations

For each participant, medical characteristics and surgical and anaesthesia data including ventilatory parameters were recorded (Supplementary Table S2). Respiratory parameters were calculated for V_T_ (using predicted body weight [PBW]), driving pressure,^12^ mechanical power of ventilation,^13^, ^14^, ^15^ and respiratory system compliance (Supplementary material).

### Endpoints

The primary endpoint in PROVHILO and PROBESE was a collapsed composite of postoperative pulmonary complications within the first five postoperative days, and were further classified by severity (Supplementary Table S3). Secondary endpoints included unplanned ICU admission, hospital length of stay, hospital mortality, and intraoperative complications such as arrhythmia, hypotension, hypoxemia, and the need for rescue manoeuvre.

### Exposure of interest

An etCO_2_ cut-off of 4.7 kPa was used to categorise patients as ‘low etCO_2_’ or ‘normal to high etCO_2_’ using the lowest intraoperative etCO_2_, as was done in previous studies.^3^^,^^6^^,^^7^

### Primary outcome

The primary outcome was the incidence of postoperative pulmonary complications compared between participants with ‘low etCO_2_’ and those with ‘normal-high etCO_2_’.

### Statistical analysis

Participant characteristics and outcome variables are presented as mean or median (interquartile range [IQR]), or number with percentage, where appropriate. Differences in baseline characteristics between ‘low etCO_2_’ and ‘normal-high etCO_2_’ patients were analysed using the Pearson χ^2^ or Fisher exact tests for categorical variables and one-way analysis of variance (anova) or Kruskal–Wallis test for continuous variables. Ventilator settings and ventilation parameters were summarised for each participant using the lowest etCO_2_ at the first hour after the start of intraoperative ventilation.

For the primary outcome, we used Fisher’s exact test and also performed a time-to-event analysis (unadjusted Cox proportional hazard model), considering mortality as a competing risk for postoperative pulmonary complications presented as Kaplan–Meier plots. We used a time-weighted analysis to determine the association of the intraoperative etCO_2_ with postoperative pulmonary complications, using a generalised mixed model. Locally estimated scatterplot smoothing (LOESS) regression was used to see the relationship between mean etCO_2_ as a continuous variable and postoperative pulmonary complications. A Box–Tidwell test was performed to determine if the linearity assumption was met. Two subgroup analyses were conducted with the primary analysis, to compare the effects of low (control) and high (intervention) PEEP, and the effect of open *vs* laparoscopic surgery. We also performed propensity score matching (Supplementary material). Because the primary outcome consists of several binary pulmonary complications, we also performed an additional analysis to investigate the hierarchy of clinical importance within the individual components of both the composite outcome and the combination of postoperative pulmonary complications and patient-centred outcomes, as a way to provide insights on the interpretation of outcomes and provide information for future trials design (Supplementary material).

Statistical significance was set at *P*<0.05. As all *post hoc* analyses should be considered exploratory, no correction for multiple testing was performed.^16^ All analyses were performed using the R statistics version 4.0.4 (Core Team, Vienna, Austria, 2021). PRISMA IPD 2015 guidelines were followed to report the results of this analysis (Supplementary material). No formal sample size calculation was performed for this reanalysis of trial data.

## Results

### Participant characteristics

Between 2011 and 2018, the two studies enrolled 2870 participants for analysis at 77 sites in 23 countries. We excluded 67 emergency surgery patients and 10 patients for missing etCO_2_ data, leaving 2793 patients for final analysis (Supplementary Fig. S1). Participants with low and normal-high etCO_2_ underwent similar types of surgery (Table 1). Participants with low etCO_2_ were older, taller, and had a lower BMI and higher ARISCAT risk scores (Table 1).

**Table 1:** Participant characteristics. The data is presented as median (IQR). APACHE, Acute Physiology and Chronic Health Evaluation; ARISCAT, Assess Respiratory Risk in Surgical Patients in Catalonia; COPD, chronic obstructive pulmonary disease; etCO_2_, end-tidal carbon dioxide; PBW, predicted body weight; SAPS, Simplified Acute Physiology Score. ∗*P*<0.05 is for statistical significance.

Characteristics	Low etCO_2_	Normal-high etCO_2_	*P*-value∗
	(*n*=821)	(*n*=1972)	
Age (yr)	62 (50–71)	51 (40–61)	<0.001
Female sex, *n* (%)	431 (52)	1291 (65)	<0.001
Height (cm)	168 (9)	166 (10)	<0.001
Weight (kg)	88 (28)	112 (30)	<0.001
PBW (kg)	61 (54–69)	59 (52–67)	<0.001
BMI (kg m^−2^)	28 (24–38)	40 (36–46)	<0.001
ARISCAT score	41 (34–42)	38 (31–41)	<0.001
ASA physical status, *n* (%)			
1	56 (7)	87 (4)	0.05
2	412 (50)	966 (49)	
3	344 (42)	885 (45)	
4	6 (1)	17 (1)	
Comorbid disease, *n* (%)			
Cancer	377 (46)	393 (20)	<0.001
Heart failure	136 (17)	105 (5)	<0.001
COPD	65 (8)	105 (5)	0.01
Surgery characteristics, *n* (%)			
Abdominal	778 (95)	1837 (93)	0.12
Non-abdominal	43 (5)	134 (7)	

### Ventilation characteristics

Compared with participants with normal-high etCO_2_, participants with low etCO_2_ had higher V_T_ and a higher minute volume normalised to body weight (Table 2), with lower mechanical power and higher respiratory system compliance, both in the overall cohort and for those allocated to receive low PEEP (Supplementary Table S4).

**Table 2:** Ventilation characteristics (after 1 h). Data is presented as median (IQR). etCO_2_, end-tidal carbon dioxide; Fio_2_, fraction of inspired oxygen score; PBW, predicted body weight; PEEP, positive end-expiratory pressure; Ppeak, peak pressure; Pplat, plateau pressure. ∗Respiratory system compliance=ratio of tidal volume to driving pressure. ^†^Pplat was available only for the PROBESE study.

Characteristics	Low etCO_2_	Normal-high etCO_2_	*P*-value
	(*n*=821)	(*n*=1972)	
Tidal volume (ml kg^−1^ PBW)	7.8 (7.1–8.1)	7.1 (7.0–7.5)	<0.001
Respiratory rate (bpm)	12 (10–14)	15 (12–18)	<0.001
<10	84 (10%)	98 (6%)	<0.001
10–15	623 (76%)	886 (45%)	
>15	112 (14%)	967 (49%)	
Fio_2_ (%)	43 (40–50)	42 (40–50)	0.95
etCO_2_ (kPa)	4.4 (4.1–4.7)	5.3 (5.1–5.9)	<0.001
Respiratory system compliance∗ (ml cmH_2_O^−1^)	36 (27–49)	30 (23–40)	<0.001
PEEP (cmH_2_O)	4 (2–12)	11 (4–12)	<0⋅001
Pplat^†^ (cmH_2_O)	22 (19–25)	23 (20–25)	0.12
Ppeak (cmH_2_O)	22 (18–26)	26 (23–30)	<0.001
Driving pressure (cmH_2_O)	14 (10–18)	15 (11–19)	0.42
Power (J min^−1^)	11 (9–14)	13 (10–16)	<0.001
Minute volume (normalised to body weight) (ml kg^−1^ min^−1^)	69 (57–83)	60 (51–69)	<0.001
<60	239 (31%)	857 (50%)	<0.001
60–80	298 (39%)	686 (41%)	
80–100	170 (22%)	134 (8%)	
>100	55 (8%)	26 (1%)	

### Primary endpoint

**Fig 1:**
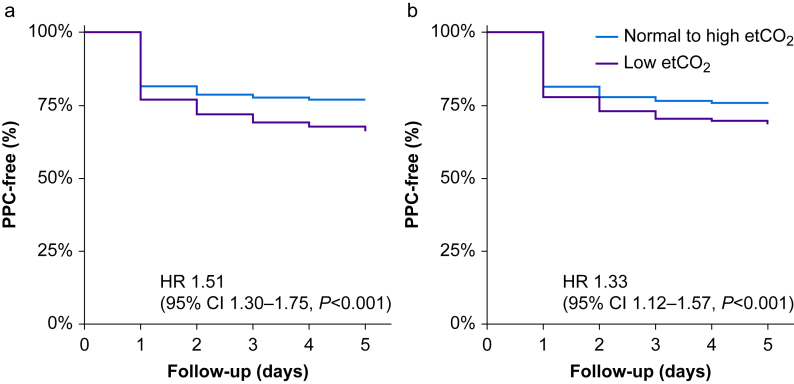
Incidence of postoperative pulmonary complications over 5 days after surgery in patients with or without hypocapnia. (a) Unadjusted cohort. (b) Propensity-matched cohort. etCO_2_, end-tidal carbon dioxide; HR, hazard ratio; 95% CI, 95% confidence interval; PPCs, postoperative pulmonary complications.

Postoperative pulmonary complications developed in 278/821 (34%) participants with low etCO_2_ (Table 3), compared with 462/1972 (23%) participants with normal-high etCO_2_ (hazard ratio [HR], 1.51 [1.3–1.75]; *P*<0.001; Fig. 1a). This difference was driven by the development of mild respiratory failure, bronchospasm, pulmonary infections, and pleural effusion (Table 3), after adjusting for confounders (32% *vs* 25%; HR, 1.33 [1.12–1.57]; *P*<0.001; Fig. 1b) and PEEP levels (Supplementary Table S5). Outcomes after open abdomen surgery were similar (Supplementary Table S6).

**Table 3:** Clinical Outcomes including postoperative pulmonary complications. ARDS, acute respiratory distress syndrome; etCO_2_, end-tidal carbon dioxide. ∗Intraoperative hypoxemia was available only for the PROBESE study. **^†^**The propensity-matched cohort was matched on age, BMI, compliance, chronic obstructive pulmonary disease, and type of surgery.

Outcome	Low etCO_2_	Normal-high etCO_2_	*P*-value
	(*n*=821)	(*n*=1972)	
Postoperative pulmonary complications, *n* (%)	278 (34)	462 (23)	<0.001
Bronchospasm	28 (3.4)	26 (1.3)	<0.001
Pulmonary infections	71 (8.6)	66 (3.3)	<0.001
Aspiration	4 (0.5)	3 (0.2)	0.11
Atelectasis	56 (6.8)	114 (5.8)	0.29
ARDS	5 (0.6)	6 (0.3)	0.24
Pleural effusion	106 (12.9)	102 (5.2)	<0.001
Mild respiratory failure	175 (21.3)	297 (15.1)	<0.001
Severe respiratory failure	64 (7.8)	122 (6.2)	0.11
Intraoperative complications, *n* (%)	539 (66)	1185 (60)	0.006
Arrhythmia	61 (7.4)	130 (6.6)	0.42
Hypotension	314 (38.2)	528 (26.8)	<0.001
Hypoxaemia∗	24 (2.9)	158 (8)	0.19
Need for rescue manoeuvre	54 (6.6)	217 (11)	<0.001
Unexpected ICU admission, *n* (%)	163 (19.9)	110 (5.6)	<0.001
Hospital length of stay,median (IQR) (days)	8 (5–13)	4 (3–7)	<0.001
Hospital mortality, *n* (%)	12 (1.5)	16 (0.8)	0.11
Propensity-matched cohort^†^	**(*n*=698)**	**(*n*=1512)**	
Postoperative pulmonary complications, *n* (%)	221 (32)	371 (25)	<0.001
Intraoperative complications, *n* (%)	452 (65)	921 (61)	0.08

### Secondary analyses

**Fig 2:**
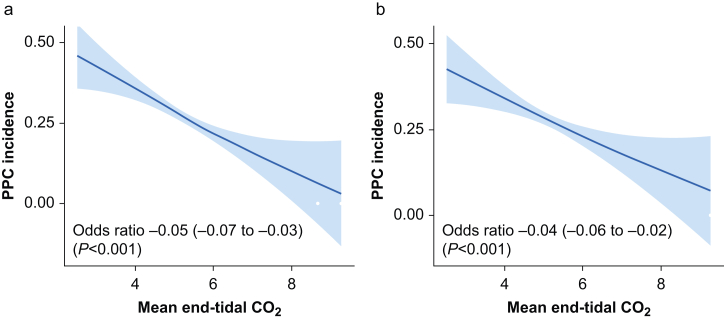
Relationship between mean end-tidal carbon dioxide (kPa) and the primary outcome. (a) Unadjusted cohort. (b) Propensity-matched cohort. Linearity of the relationship was confirmed using the Box–Tidwell test (*P*=0.75). PPCs, postoperative pulmonary complications.

There was an inverse linear relationship between the mean etCO_2_ and postoperative pulmonary complications (Fig. 2, Supplementary Fig. S2). The propensity-matched analysis (Supplementary Fig. S3) confirmed the findings of the unmatched analysis (Table 3). Low etCO_2_ was associated with more frequent unplanned admissions to ICU and longer hospital length of stay (Table 3).

### Sensitivity analysis

Postoperative pulmonary complications were more frequent in participants with low etCO_2,_ irrespective of BMI (Table 4). We found no interaction between PEEP (*P*=0.99) or ARISCAT (*P*=0.42) and the development of postoperative pulmonary complications.

**Table 4:** Sensitivity analysis for postoperative pulmonary complications. Obesity: BMI ≥30 kg m^−2^. *P*<0.05 is significant. etCO_2_, end-tidal carbon dioxide; PPCs, postoperative pulmonary complications.

Outcome	Low etCO_2_	Normal-high etCO_2_	*P*-value
Obese patients with PPCs	108 (29%)	365 (23%)	0.007
Non-obese patients with PPCs	170 (38%)	97 (27%)	0.002

### Win ratio analysis

There was no difference in wins or losses in the pulmonary endpoints (0.60; 95% confidence interval [CI], 0.51–0.70; Supplementary Fig. S4). The overall win ratio analysis of a hierarchical order of patient-centred outcomes in 1 584 916 combinations was worse in participants with low etCO_2_ compared with participants with normal-high etCO_2_ (0.37; 95% CI, 0.32–0.41; Supplementary Fig. S5).

## Discussion

In this *post hoc* individual-level meta-analysis of two large RCTs in patients undergoing general anaesthesia for surgery, trial participants with low etCO_2_ had an increased risk of postoperative pulmonary complications, despite the use of a lung-protective intraoperative ventilation strategy. In addition, trial participants randomised to receive lower PEEP were more likely to have low etCO_2_. There was an inverse relationship between the mean etCO_2_ and postoperative pulmonary complications. In our analysis, we found that mild respiratory failure or hypoxemia was higher in the low etCO_2_ group, and was observed in nearly one-fifth of patients. Bronchospasm, pleural effusion, and pulmonary infection were also more common in patients with low etCO_2_.

Our findings are in line with previous studies. Hypocapnia in mechanically ventilated patients has been attributed to excessive alveolar ventilation. Although the protocol in both RCTs mandated a low V_T_ for lung-protective ventilation (LPV) with a target of ≤8 ml kg^−1^ PBW, low etCO_2_ was seen in 29% of the patients. Previous observational studies also reported a high incidence of hypocapnia, between 29% and 66%, during general anaesthesia.^2^^,^^5^ The protocolised intraoperative ventilation limits the settings of V_T_, Pplat, and PEEP. However, titration of the RR was at the discretion of the anaesthesiologists to keep etCO_2_ in the range of 4.7–6.0 kPa.^7^^,^^8^^,^^17^ The use of relatively higher V_T_ and a possible reluctance to reduce the RR in compliant lungs despite low levels of etCO_2_ may explain the inadvertent hyperventilation and resultant low etCO_2_.

In our previous *post hoc* analysis of the LAS VEGAS (Local ASsessment of Ventilatory management during General Anaesthesia for Surgery) database, a prospective observational study, there was no difference in overall postoperative pulmonary complications between low and normal-high etCO_2_ patients. However, the incidence of severe postoperative pulmonary complications was higher in low etCO_2_ patients, with an inverse dose-dependent relationship.^18^ Few other studies also described an association between intraoperative hypocapnia and worse patient outcomes, such as mortality and increased length of stay.^5^, ^6^, ^7^ The present study was not designed to identify the potential sources of the increased incidence of postoperative pulmonary complications. It is noteworthy that mechanical power, a surrogate of lung strain, was lower in patients with low etCO_2_. This discrepancy may be explained by the difference in respiratory system compliance between low etCO_2_ patients and those with a normal-high etCO_2_. However, certain ventilator settings associated with the lower etCO_2_ group––particularly the use of higher V_T_ and increased minute volume normalised to body weight––may have contributed to the higher incidence of postoperative pulmonary complications observed in this group, which merits further exploration.

The primary endpoint of the two trials was a composite of postoperative pulmonary complications. However, comparing heterogeneous components can be challenging because of the frequency, treatment effects, and clinical significance of individual outcomes.^19^ The win ratio analysis using hierarchical outcomes did not find a difference in postoperative pulmonary complications, but the analysis including patient-centred outcomes showed a less favourable outcome in patients with low etCO_2_ compared with those with high or normal etCO_2_. In the hierarchical order, this result appears to be driven by a higher hospital mortality and longer hospital length of stay in patients with low etCO_2_, without a meaningful difference in the defined pulmonary endpoints. This mirrors the results of the primary analysis, save for the absence of difference in mortality between the groups.

Expiratory CO_2_ is a surrogate for alveolar levels, as Paco_2_ could be higher than etCO_2_ by 0.3–0.7 kPa. This gradient between Paco_2_ and etCO_2_ primarily reflects alveolar dead space. The gradient may widen further in the presence of ventilation–perfusion mismatch, elevated PEEP, pulmonary pathology, or low right-sided cardiac output. Errors in etCO_2_ measurement techniques can also contribute to discrepancies. In patients undergoing mechanical ventilation during general anaesthesia, these factors––particularly increased dead space and impaired perfusion––are key contributors to the Paco_2_–etCO2 gap.^20^, ^21^, ^22^ The sensitivity analysis suggested PEEP did affect the primary endpoints of this study. However, in the absence of arterial blood gas measurements, it remains possible that etCO_2_ underestimated Paco_2_ in some patients, and thus did not accurately reflect hypocapnia. Nevertheless, a strong correlation has been observed in mechanically ventilated patients, especially in those with healthy lungs.^20^^,^^21^ Additionally, the association between inadvertent hyperventilation and adverse postoperative outcomes, including postoperative pulmonary complications, is consistent with findings from other studies that included direct Paco_2_ measurements.^23^^,^^24^ Low cardiac output reduces pulmonary perfusion and increases the Paco_2_–etCO_2_ gradient.^21^^,^^22^ Notably, we observed a higher incidence of hypotension in patients with low etCO_2_ compared with those with normal-high etCO_2_. However, in the absence of cardiac output measurements, it remains unclear whether hypotension was a consequence of inadvertent hyperventilation and resulting hypocapnia or whether it contributed to a lower etCO_2_ and widened Paco_2_–etCO_2_ gradient.

The physiological impact of hypocapnia in humans is diverse, affecting multiple organs, and is not fully understood. Among its most pronounced effects, hypocapnia can reduce cerebral blood flow (CBF) and increase cerebral oxygen consumption, thereby dissociating the relation of metabolism to CBF.^25^ In a recent study, pre-hospital hypocapnia (defined by etCO_2_ <4.7 kPa) was associated with lower survival in patients with severe traumatic brain injury.^26^ Hypocapnia can adversely impact ventilation–perfusing matching, mediated by various mechanisms such as reducing respiratory system compliance, attenuating hypoxic pulmonary vasoconstriction, bronchospasm, and intrapulmonary shunting.^26^, ^27^, ^28^ In an isolated rat lung model, severe hypocapnia was associated with an increased risk of pulmonary oedema, because it impaired alveolar fluid resorption.^29^ Further, hypocapnia was linked to cardiac arrhythmias and reduced stroke volume and tissue oxygen delivery in experimental animals.^30^ The evidence suggesting potential harm in this patient-level meta-analysis of prospective studies is compelling; however, it is important to note that this analysis is *post hoc*. Therefore, it is imperative that future prospective studies validate the advantages of utilising intraoperative LPV that targets normal to elevated levels of etCO_2_.

Our study has several strengths. This is the first individual patient-level meta-analysis involving multi-centric large cohorts with a strict analysis plan to evaluate the relationship between low etCO_2_ levels and postoperative pulmonary complications. The studies included in this analysis used a protocolised LPV strategy with low V_T_ and lower Pplat, minimising the impact of high V_T_ on lung injury.^8^^,^^9^ Additionally, we explored the dose-dependent relationship between etCO_2_ and postoperative pulmonary complications. Finally, the propensity-matched cohorts mitigated the confounding effect of known covariates. This study also has several limitations. First, the merged databases included two previous RCTs focused on the effect of intraoperative PEEP on postoperative pulmonary complications. Hence, the relationship between hypocapnia and postoperative pulmonary complications can only be interpreted as an association and not a causal relationship. It is hypothesis-generating at best but shows a clear signal which should be confirmed in a prospective trial. Second, etCO_2_ was used as a surrogate marker for hypocapnia in this *post hoc* analysis. However, without concurrent measurements of cardiac output and Paco_2_, it is plausible that some patients had a substantial Paco_2_–etCO_2_ gradient, and that not all patients classified as having low etCO_2_ were truly hypocapnic. Moreover, the higher incidence of hypotension observed in low etCO_2_ group may have influenced the results, potentially through its impact on pulmonary perfusion and the etCO_2_ values.^30^^,^^31^ However, the linear association observed in the LOESS plots provides a clear signal that lower CO_2_ is associated with more postoperative pulmonary complications. Third, we dichotomised CO_2_ levels to enhance clinical interpretability, reflecting threshold-based decision-making common in practice. The cut-off was informed by previous literature.^3^^,^^6^^,^^7^ Although continuous modelling (e.g. with splines) may capture non-linear associations, the skewed distribution in our dataset raised concerns about overfitting and instability. Dichotomisation provided a parsimonious, robust approach suitable for our sample size. We acknowledge the trade-off in information loss and recommend future studies explore continuous modelling in larger datasets**.** Finally, although emergency surgery was excluded in this study, recruited patients in both RCTs were at high risk for PPCs. Hence, the increased risk of postoperative pulmonary complications observed with low etCO_2_ reflects systemic hypocapnia, which needs to be verified in future studies.

In summary, this individual patient-level meta-analysis of two RCTs on LPV undergoing general anaesthesia found that intraoperative low etCO_2_ (<4.7 kPa) was associated with a higher rate of postoperative pulmonary complications, with a dose-dependent inverse relationship between low etCO_2_ and pulmonary pathology.

## Authors’ contributions

Wrote the first draft of the manuscript: PN, SNTH

Contributed to the conception and design, acquisition of data, or analysis and interpretation of data, to the final drafting of the article: PN

Performed the statistical analysis, acquisition of data, or analysis and interpretation of data: DMPM

Revised the manuscript critically for important intellectual content: SNTH

Contributed to conception and design and revised it critically for important intellectual content: FP, CF, TB, MGA, LB, SMB, PS, ASN, MJS

Read and approved the submitted manuscript and agreed to be accountable for all aspects of the work and thereby, ensuring that questions related to the accuracy or integrity of any part of the work are appropriately investigated and resolved: all authors

## Data availability statement

The dataset used and analysed during this study are available from the corresponding author upon reasonable request.

## Funding

European Society of Anaesthesiology and Academical Medical Center (Amsterdam, The Netherlands) (for the PROVHILO trial); Clinical Trials Network of the European Society of Anaesthesiology; Technische Universität Dresden; Conselho Nacional de Desenvolvimento Científico e Tecnológico; Association of Anaesthetists of Great Britain and Ireland and the Northern Ireland Society of Anaesthetists (for the PROBESE trial).

## Declarations of interest

The authors declare that they have no conflicts of interest.
