# Supplementary material for: Association of intraoperative end-tidal CO2 levels with postoperative outcomes: a patient-level analysis of two randomised clinical trials
Source: Br J Anaesth. 2025 Aug 14;135(6):1761–9. doi: 10.1016/j.bja.2025.07.076 (PMC12799414; doi:10.1016/j.bja.2025.07.076)

**SUPPLEMENTARY DATA:** Associations of Intraoperative Hypocapnia with Postoperative Outcome––a patient–level analysis of two randomized clinical trials.

Nasa et al

**Contents list**

Contents

[1 List of Collaborators 2](#_Toc205914317)

[**1.1** **The REPEAT INVESTIGATORS** 2](#_Toc205914318)

[**1.2** **THE PROVHILO INVESTIGATORS** 3](#_Toc205914319)

[**1.3** **THE iPROVE INVESTIGATORS** 6](#_Toc205914320)

[**1.4** **THE PROBESE INVESTIGATORS** 11](#_Toc205914321)

[2 PRISMA-IPD Checklist of items to include when reporting a systematic review and meta-analysis of individual participant data (IPD) 23](#_Toc205914322)

[3 Supplementary Methods: respiratory parameters – calculations. 27](#_Toc205914323)

[4 Supplementary Methods: Propensity score matching 27](#_Toc205914324)

[5 Hierarchy of clinical importance analysis 28](#_Toc205914325)

[6 Supplementary Table 1: Study characteristics of the PROVHILO and PROBESE 29](#_Toc205914326)

[7 Supplementary Table 2: Data collected in the original studies of the PROVHILO and PROBESE 32](#_Toc205914327)

[8 Supplementary Table 3: Definitions of the outcomes in the original study 34](#_Toc205914328)

[9 Supplementary Table 4. Ventilation characteristics (after one hour) 36](#_Toc205914329)

[10 Supplementary Table 5. Outcome in low and high PEEP groups 37](#_Toc205914330)

[11 Supplementary Table 6. Outcome in open abdomen and laparoscopic surgical approach 38](#_Toc205914331)

[12 Supplementary Figure 1: Consort diagram displaying the included patients in the study 39](#_Toc205914332)

[13 Supplementary Figure 2: Mean etCO_2_ plotted against the log odds (for PPCs) 40](#_Toc205914333)

[14 Supplementary Figure 3: Love plot for assessing balance of covariates 41](#_Toc205914334)

[15 Supplementary Figure 4: Distribution of Wins, Ties and Losses on pulmonary endpoints among normal to high etCO2 and low etCO2 patients 42](#_Toc205914335)

[16 Supplementary Figure 5: Distribution of Wins, Ties and Losses on overall hierarchical endpoints among normal to high etCO2 and low etCO2 patients 43](#_Toc205914336)

# List of Collaborators

## **The REPEAT INVESTIGATORS**

*Steering and executive committees*

| Niklas S Campos | Hospital Israelita Albert Einstein,  Instituto do Coração, Hospital das Clinicas HCFMUSP, Faculdade de Medicina, Universidade de Sao Paulo, São Paulo, Brazil |
| --- | --- |
| Thomas Bluth | University Hospital Carl Gustav Carus, Technische Universität Dresden, Dresden, Germany |
| Sabrine NT Hemmes | Amsterdam UMC, University of Amsterdam, Amsterdam, The Netherland |
| Julian Librero | Red de Investigación en Servicios de Salud en Enfermedades Crónicas (REDISSEC), Pamplona, Spain |
| Natividad Pozo | Hospital Clinic de Barcelona, Barcelona, Spain |
| Carlos Ferrando | CIBER of Respiratory Disease, Instituto de Salud Carlos III, Madrid, Spain  IRCCS San Martino Policlinico Hospital, Genoa, Italy |
| Lorenzo Ball | IRCCS San Martino Policlinico Hospital, University of Genoa, Genoa, Italy |
| Guido Mazzinari | Hospital de Manises, Instituto de Investigación Sanitaria La Fe Valencia, Spain |
| Paolo Pelosi | IRCCS San Martino Policlinico Hospital, University of Genoa, Genoa, Italy |
| Marcelo Gama de Abreu | University Hospital Carl Gustav Carus, Technische Universität Dresden, Dresden, Germany |
| Marcus J Schultz | Amsterdam UMC, University of Amsterdam, Amsterdam, The Netherland  Mahidol University, Bangkok, Thailand |
| Ary Serpa Neto | Hospital Israelita Albert Einstein, Instituto do Coração, Hospital das Clinicas HCFMUSP, Faculdade de Medicina, Universidade de Sao Paulo, São Paulo, Brazil  Amsterdam UMC, University of Amsterdam, Amsterdam, The Netherland |

## **THE PROVHILO INVESTIGATORS**

*Steering and executive committees*

| Sabrine NT Hemmes | Amsterdam UMC, University of Amsterdam, Amsterdam, The Netherland |
| --- | --- |
| Marcelo Gama de Abreu | University Hospital Carl Gustav Carus, Technische Universität Dresden, Dresden, Germany |
| Paolo Severgnini | University of Insubria, Varese, Italy |
| Markus W Hollmann | Amsterdam UMC, University of Amsterdam, Amsterdam, The Netherland |
| Jan M Binnekade | Amsterdam UMC, University of Amsterdam, Amsterdam, The Netherland |
| Hermann Wrigge | University of Leipzig, Leipzig, Germany |
| Jaume Canet | Hospital Universitari Germans Trias I Pujol, Barcelona, Spain |
| Michael Hiesmayr | Medical University Vienna, Vienna, Austria |
| Werner Schmid | Medical University Vienna, Vienna, Austria |
| Edda Tschernko | Medical University Vienna, Vienna, Austria |
| Samir Jaber | Saint Eloi University Hospital, Montpellier, France |
| Göran Hedenstierna | Uppsala University, Uppsala, Sweden |
| Christian Putensen | University Hospital Bonn, Bonn, Germany |
| Paolo Pelosi | IRCCS San Martino Policlinico Hospital, University of Genoa, Genoa, Italy |
| Marcus J Schultz | Amsterdam UMC, University of Amsterdam, Amsterdam, The Netherland  Mahidol University, Bangkok, Thailand |

*Investigators*

| (**, indicates local principal investigators; names are listed in alphabetical order*) | |
| --- | --- |
| Agnes Marti | Hospital Universitari Germans Trias I Pujol, Spain |
| Alessandro Bacuzzi | University of Insubria - Azienda Ospedaliera Fondazione Macchi - Ospedale di Circolo, Italy |
| Alexander Brodhun | Johannes Gutenberg - Universität Mainz, Germany |
| Alexandre Molin* | Università degli Studi di Genova, IRCCS San Martino IST, Italy |
| Alfred Merten | Hospital Sant Pau, Spain |
| Ana Parera | Hospital Sant Pau, Spain |
| Andrea Brunelli* | Hospital Universitari Germans Trias I Pujol, Spain |
| Andrea Cortegiani | Università degli Studi di Palermo, Italy |
| Andreas Güldner | University Hospital Dresden, Technische Universität Dresden, Germany |
| Andreas W Reske | University of Leipzig, Germany |
| Angelo Gratarola | Università degli Studi di Genova, IRCCS San Martino IST, Italy |
| Antonino Giarratano* | Università degli Studi di Palermo, Italy |
| Bea Bastin | Düsseldorf University Hospital, Heinrich-Heine University Düsseldorf, Germany |
| Bjorn Heyse | Ghent University Hospital, Belgium |
| Branka Mazul-Sunko* | University Hospital Sveti Duh, Croatia |
| Bruno Amantea | University "Magna Graecia" of Catanzaro, Italy |
| Bruno Barberis | Azienda Sanitaria Locale TO3 - Ospedale di Rivoli, Italy |
| Christian Putensen* | University Hospital of Bonn Medical School, Germany |
| Christopher Uhlig | University Hospital Dresden, Technische Universität Dresden, Germany |
| Conrado Minguez Marín | Consorcio Hospital General Universitario Valencia, Spain |
| Cristian Celentano | Azienda Sanitaria Locale TO3 - Ospedale di Rivoli, Italy |
| Daniela La Bella | University of Foggia, Italy |
| David D’Antini | University of Foggia, Italy |
| David Velghe* | ZNA Middelheim, Belgium |
| Demet Sulemanji | Massachusetts General Hospital, USA |
| Edoardo De Robertis* | University of Napoli Federico II, Italy |
| Eric Hartmann | Johannes Gutenberg - Universität Mainz, Germany |
| Francesca Montalto | Università degli Studi di Palermo, Italy |
| Francesco Tropea | University "Magna Graecia" of Catanzaro, Italy |
| Gary H Mills* | Sheffield Teaching Hospitals, United Kingdom |
| Gilda Cinnella* | University of Foggia, Italy |
| Giorgio Della Rocca* | Università degli Studi di Udine, Italy |
| Girolamo Caggianelli | University of Foggia, Italy |
| Giulia Pellerano | Università degli Studi di Genova, IRCCS San Martino IST, Italy |
| Giuseppina Mollica | University of Foggia, Italy |
| Guillermo Bugedo* | Hospital Clínico de la Pontificia Universidad Católica de Chile, Chile |
| Hermann Wrigge* | University of Leipzig, Germany |
| Jan-Paul Mulier* | AZ St Jan, Belgium |
| Jeroen Vandenbrande | Virga Jesse Ziekenhuis, Belgium |
| Johann Geib | Düsseldorf University Hospital, Heinrich-Heine University Düsseldorf, Germany |
| Jonathan Yaqub | University Hospital Dresden, Technische Universität Dresden, Germany |
| Jorge Florez | Hospital Clínico de la Pontificia Universidad Católica de Chile, Chile |
| Juan F Mayoral | Fundación Puigvert, Spain |
| Juraj Sprung* | Mayo Clinic, USA |
| Jurgen Van Limmen | Ghent University Hospital, Belgium |
| Lieuwe DJ Bos | Academic Medical Center, University of Amsterdam, The Netherlands |
| Luc de Baerdemaeker | Ghent University Hospital, Belgium |
| Luc Jamaer* | Virga Jesse Ziekenhuis, Belgium |
| Luigi Spagnolo* | Azienda Sanitaria Locale TO3 - Ospedale di Rivoli, Italy |
| Lydia Strys | Johannes Gutenberg - Universität Mainz, Germany |
| Manuel Granell Gil* | Consorcio Hospital General Universitario Valencia, Spain |
| Marcelo Gama de Abreu* | University Hospital Dresden, Technische Universität Dresden, Germany |
| Marcos F Vidal Melo* | Massachusetts General Hospital, USA |
| Marcus J Schultz* | Academic Medical Center, University of Amsterdam, The Netherlands |
| Maria Carmen Unzueta* | Hospital Sant Pau, Spain |
| Maria Victoria Moral | Hospital Sant Pau, Spain |
| Marion Ferner | Johannes Gutenberg - Universität Mainz, Germany |
| Markus W Hollmann | Academic Medical Center, University of Amsterdam, The Netherlands |
| Martin Weiss | Düsseldorf University Hospital, Heinrich-Heine University Düsseldorf, Germany |
| Massimo Vanoni | University of Insubria - Azienda Ospedaliera Fondazione Macchi - Ospedale di Circolo, Italy |
| Maximilian S Schaefer | Düsseldorf University Hospital, Heinrich-Heine University Düsseldorf, Germany |
| Mercè Prieto | Fundación Puigvert, Spain |
| Michele Grio | Azienda Sanitaria Locale TO3 - Ospedale di Rivoli, Italy |
| Paolo Severgnini* | University of Insubria - Azienda Ospedaliera Fondazione Macchi - Ospedale di Circolo, Italy |
| Peter Markus Spieth | University Hospital Dresden, Technische Universität Dresden, Germany |
| Philipp Simon | University of Leipzig, Germany |
| Phoebe Bodger* | Barts Health NHS Trust, United Kingdom |
| Pilar Sierra | Fundación Puigvert, Spain |
| Rita Laufenberg-Feldmann* | Johannes Gutenberg - Universität Mainz, Germany |
| Roberta Rusca | Università degli Studi di Genova, IRCCS San Martino IST, Italy |
| Rodolfo Proietti* | Università degli Studi di Roma Cattolica, Italy |
| Sabrine NT Hemmes | Academic Medical Center, University of Amsterdam, The Netherlands |
| Santi Maurizio Raineri | Università degli Studi di Palermo, Italy |
| Santo Caroleo* | University "Magna Graecia" of Catanzaro, Italy |
| Sergi Sabaté* | Fundación Puigvert, Spain |
| Stefan De Hert* | Ghent University Hospital, Belgium |
| Stefano Pezzato | Università degli Studi di Genova, IRCCS San Martino IST, Italy |
| Tanja A Treschan* | Düsseldorf University Hospital, Heinrich-Heine University Düsseldorf, Germany |
| Tatjana Goranovic | University Hospital Sveti Duh, Croatia |
| Thea Koch | University Hospital Dresden, Technische Universität Dresden, Germany |
| Thomas Bluth | University Hospital Dresden, Technische Universität Dresden, Germany |
| Thomas Kiss | University Hospital Dresden, Technische Universität Dresden, Germany |
| Valter Perilli | Università degli Studi di Roma Cattolica, Italy |
| Virginia Cegarra | Hospital Sant Pau, Spain |
| Werner Schmid* | Medical University Viena, Viena |

*Information and funding*

The PROVHILO trial is a collaboration of the Protective Ventilation Network (PROVENet). The trial was funded by the European Society of Anaesthesiology (ESA) and the Academical Medical Center (AMC, Amsterdam, The Netherlands).

## **THE iPROVE INVESTIGATORS**

*Steering and executive committees*

| Carlos Ferrando | Hospital Clinic de Barcelona, Barcelona, CIBER of Respiratory Disease, Instituto de Salud Carlos III, Madrid Spain |
| --- | --- |
| Javier Belda | Medicine Faculty, Universidad de Valencia, Valencia, Spain |
| Marina Soro | Hospital Clínico Universitario de Valencia, Valencia, Spain |
| Jaume Canet | Hospital Universitario Germans Tries i Pujol, Barcelona, Spain |
| Carmen Unzueta | Hospital Universitario San Pau, Barcelona, Spain |
| Fernando Suarez-Sipmann | Hospital Universitario La Princesa, Madrid, Spain |
| Julián Librero | Red de Investigación en Servicios de Salud en Enfermedades Crónicas (REDISSEC), Pamplona, Spain |
| Alicia Llombart | Corachan Hospital, Barcelona, Spain |
| Lucas Rovira | Hospital General Universitario de Valencia, Valencia, Spain |
| Manuel Granell | Hospital General Universitario de Valencia, Valencia, Spain |
| César Aldecoa | Hospital Universitario Río Hortega, Valladolid, Spain |
| Oscar Diaz-Cambronero | Hospital Universitario y Politécnico La Fe, Valencia, Spain |
| Jaume Balust | Hospital Clínic i Provincial Universitario de Barcelona, Barcelona, Spain |
| Ignacio Garutti | Hospital General Universitario Gregorio Marañón, Madrid, Spain |
| Rafael Gonzalez | Hospital General Universitario Gregorio Marañón, Madrid, Spain |
| Lucia Gallego | Hospital Universitario Miguel Servet de Zaragoza, Zaragoza, Spain |
| Santiago Garcia del Valle | Hospital Fundación de Alcorcón, Alcorcón, Spain |
| Javier Redondo | Hospital General Universitario de Ciudad Real, Ciudad Real, Spain |
| David Pestaña | Hospital Universitario Ramón y Cajal, Madrid, Spain |
| Aurelio Rodríguez | Hospital de Gran Canaria, Dr. Negrín, Gran Canaria, Spain |
| Javier García | Hospital Universitario Puerta de Hierro de Majadahonda, Madrid, Spain |
| Manuel de la Matta | Hospital Universitario Virgen del Rocio de Sevilla, Sevilla, Spain |
| Maite Ibáñez | Hospital de la Marina Baixa de la Vila Joiosa, Alicante, Spain |
| Francisco Barrios | Hospital Principe de Asturias, Madrid, Spain |
| Samuel Hernández | Hospital NS de Candelaria, Santa Cruz de Tenerife, Spain |
| Vicente Torres | Hospital Son Espases de Mallorca, Mallorca, Spain |
| Salvador Peiró | Hospital Universitario y Politécnico La Fe, Valencia, Spain |
| Natividad Pozo | INCLIVA, Biomedical Research Institute, Valencia, Spain |

*Investigators*

| (*names are listed in alphabetical order*) | |
| --- | --- |
| Abigail Villena | Hospital Clínico Universitario de Valencia, Valencia, Spain |
| Albert Carramiñana | Hospital Universitario Germans Tries i Pujol, Badalona, Spain |
| Alberto Gallego-Casilda | Hospital Universitario Miguel Servet de Zaragoza, Zaragoza, Spain |
| Alejandro Duca | Hospital Clínico Universitario de Valencia, Valencia, Spain |
| Amalia Alcón | Hospital Clínic i Provincial Universitario de Barcelona, Barcelona, Spain |
| Amanda Miñana | Hospital Clínico Universitario de Valencia, Valencia, Spain |
| Ana Asensio | Hospital Universitario Miguel Servet de Zaragoza, Zaragoza, Spain |
| Ana Colás | Hospital Universitario Miguel Servet de Zaragoza, Zaragoza, Spain |
| Ana Isabel Galve | Hospital General Universitario Gregorio Marañón, Madrid, Spain |
| Ana Izquierdo | Hospital Clínico Universitario de Valencia, Valencia, Spain |
| Ana Jurado | Hospital Clínico Universitario de Valencia, Valencia, Spain |
| Ana María Pérez | Hospital de León, León, Spain |
| Ana Mugarra | Hospital Clínico Universitario de Valencia, Valencia, Spain |
| Ana Parera | Hospital Universitario San Pau, Barcelona, Spain |
| Andrea Brunelli | Hospital Universitario Germans Tries i Pujol, Badalona, Spain |
| Andrea Gutierrez | Hospital Clínico Universitario de Valencia, Valencia, Spain |
| Ángeles De Miguel | Hospital General Universitario Gregorio Marañón, Madrid, Spain |
| Angels Lozano | Hospital Clínico Universitario de Valencia, Valencia, Spain |
| Antonio Katime | Hospital Clínico Universitario de Valencia, Valencia, Spain |
| Antonio Romero | Hospital Universitario Puerta de Hierro de Majadahonda, Madrid, Spain |
| Beatriz Garrigues | Hospital Clínico Universitario de Valencia, Valencia, Spain |
| Begoña Ayas | Hospital Universitario y Politécnico La Fe, Valencia, Spain |
| Blanca Arocas | Hospital Clínico Universitario de Valencia, Valencia, Spain |
| Carlos Delgado | Hospital Clínico Universitario de Valencia, Valencia, Spain |
| Carmen Fernández | Hospital General Universitario Gregorio Marañón, Madrid, Spain |
| Carolina Romero | Hospital Clínico Universitario de Valencia, Valencia, Spain |
| Clara Gallego | Hospital Universitario Ramón y Cajal, Madrid, Spain |
| Cristina Garcés | Hospital Universitario Miguel Servet de Zaragoza, Zaragoza, Spain |
| Cristina Lisbona | Hospital General Universitario Gregorio Marañón, Madrid, Spain |
| Cristina Parrilla | Hospital Clínico Universitario de Valencia, Valencia, Spain |
| Daniel López-Herrera | Hospital Universitario Virgen del Rocio de Sevilla, Sevilla, Spain |
| Domingo González | Hospital Universitario Virgen del Rocio de Sevilla, Sevilla, Spain |
| Eduardo Llamazares | Hospital General Universitario Gregorio Marañón, Madrid, Spain |
| Elena Del Rio | Hospital Clínico Universitario de Valencia, Valencia, Spain |
| Elena Lozano | Hospital de la Marina Baixa de la Vila Joiosa, Alicante, Spain |
| Ernesto Pastor | Hospital Clínico Universitario de Valencia, Valencia, Spain |
| Estefanía Chamorro | Hospital General Universitario Gregorio Marañón, Madrid, Spain |
| Estefanía Gracia | Hospital Clínico Universitario de Valencia, Valencia, Spain |
| Ester Sánchez | Hospital de la Marina Baixa de la Vila Joiosa, Alicante, Spain |
| Esther Romero | Hospital Clínico Universitario de Valencia, Valencia, Spain |
| Fernando Díez | Hospital de León, León, Spain |
| Ferran Serralta | Hospital Clínico Universitario de Valencia, Valencia, Spain |
| Francisco Daviu | Hospital General Universitario Gregorio Marañón, Madrid, Spain |
| Francisco Sandín | Hospital Universitario Miguel Servet de Zaragoza, Zaragoza, Spain |
| Gerardo Aguilar | Hospital Clínico Universitario de Valencia, Valencia, Spain |
| Gerardo Tusman | Hospital Privado de Comunidad, Mar del Plata, Buenos Aires, Argentina |
| Gonzalo Azparren | Hospital Universitario San Pau, Barcelona, Spain |
| Graciela Martínez-Pallí | Hospital Clínic i Provincial Universitario de Barcelona, Barcelona, Spain |
| Guido Mazzinari | Hospital de Manises, Valencia, Spain |
| Inmaculada Benítez | Hospital Universitario Virgen del Rocio de Sevilla, Sevilla, Spain |
| Inmaculada Hernandéz | Hospital General Universitario Gregorio Marañón, Madrid, Spain |
| Inmaculada India | Hospital Universitario San Pau, Barcelona, Spain |
| Irene León | Hospital Clínic i Provincial Universitario de Barcelona, Barcelona, Spain |
| Isabel Fuentes | Hospital Clínico Universitario de Valencia, Valencia, Spain |
| Isabel Ruiz | Hospital Universitario Ramón y Cajal, Madrid, Spain |
| Jaume Puig | Hospital Clínico Universitario de Valencia, Valencia, Spain |
| Javie Ignacio Román | Hospital Son Espases de Mallorca, Mallorca, Spain |
| Jesús Acosta | Hospital Universitario Virgen del Rocio de Sevilla, Sevilla, Spain |
| Jesús Rico-Feijoo | Hospital Universitario Río Hortega, Valladolid, Spain |
| Jonathan Olmedo | Hospital General Universitario Gregorio Marañón, Madrid, Spain |
| Jose A. Carbonell | Hospital Clínico Universitario de Valencia, Valencia, Spain |
| Jose M. Alonso | Hospital Clínico Universitario de Valencia, Valencia, Spain |
| Jose María Pérez | Hospital General Universitario Gregorio Marañón, Madrid, Spain |
| Jose Miguel Marcos | Hospital de León, León, Spain |
| Jose Navarro | Hospital Clínico Universitario de Valencia, Valencia, Spain |
| Jose Valdivia | Hospital de la Marina Baixa de la Vila Joiosa, Alicante, Spain |
| Juan Carrizo | Hospital Clínico Universitario de Valencia, Valencia, Spain |
| Laura Piqueras | Hospital Clínico Universitario de Valencia, Valencia, Spain |
| Laura Soriano | Hospital General Universitario Gregorio Marañón, Madrid, Spain |
| Laura Vaquero | Hospital Universitario Río Hortega, Valladolid, Spain |
| Lisset Miguel | Hospital Universitario Ramón y Cajal, Madrid, Spain |
| Lorena Muñoz | Hospital General Universitario de Valencia, Valencia, Spain |
| Lucia Valencia | Hospital de Gran Canaria, Dr. Negrín, Gran Canaria, Spain |
| Luis Olmedilla | Hospital General Universitario Gregorio Marañón, Madrid, Spain |
| M^a^ Justina Etulain | Hospital General Universitario Gregorio Marañón, Madrid, Spain |
| Manuel Tisner | Hospital General Universitario Gregorio Marañón, Madrid, Spain |
| María Barrio | Hospital General Universitario Gregorio Marañón, Madrid, Spain |
| María Dolores Alonso | Hospital General Universitario de Valencia, Valencia, Spain |
| María García | Hospital Universitario Río Hortega, Valladolid, Spain |
| María J. Hernández | Hospital General Universitario de Valencia, Valencia, Spain |
| María José Alberola | Hospital Universitario y Politécnico La Fe, Valencia, Spain |
| María Parra | Hospital Clínico Universitario de Valencia, Valencia, Spain |
| María Pilar Argente | Hospital Universitario y Politécnico La Fe, Valencia, Spain |
| María Vila | Hospital Universitario y Politécnico La Fe, Valencia, Spain |
| Mario De Fez | Hospital Clínico Universitario de Valencia, Valencia, Spain |
| Marta Agilaga | Hospital Universitario San Pau, Barcelona, Spain |
| Marta Gine | Hospital Universitario San Pau, Barcelona, Spain |
| Mercedes Ayuso | Hospital Principe de Asturias, Madrid, Spain |
| Mercedes García | Hospital Universitario San Pau, Barcelona, Spain |
| Natalia Bejarano | Hospital General Universitario de Ciudad Real, Ciudad Real, Spain |
| Natalia Peña | Hospital Universitario Miguel Servet de Zaragoza, Zaragoza, Spain |
| Nazario Ojeda | Hospital de Gran Canaria, Dr. Negrín, Gran Canaria, Spain |
| Nilda Martínez | Hospital Universitario Ramón y Cajal, Madrid, Spain |
| Nuria García | Hospital Universitario y Politécnico La Fe, Valencia, Spain |
| Oto Padrón | Hospital de Gran Canaria, Dr. Negrín, Gran Canaria, Spain |
| Pablo García | Hospital Fundación de Alcorcón, Alcorcón, Spain |
| Paola Valls | Hospital Clínico Universitario de Valencia, Valencia, Spain |
| Patricia Cruz | Hospital General Universitario Gregorio Marañón, Madrid, Spain |
| Patricia Piñeiro | Hospital General Universitario Gregorio Marañón, Madrid, Spain |
| Pedro Charco | Hospital Clínico Universitario de Valencia, Valencia, Spain |
| Rafael Anaya | Hospital Universitario San Pau, Barcelona, Spain |
| Ramiro López | Hospital General Universitario Gregorio Marañón, Madrid, Spain |
| Rayco Rodríguez | Hospital de Gran Canaria, Dr. Negrín, Gran Canaria, Spain |
| Rocío Martínez | Hospital General Universitario Gregorio Marañón, Madrid, Spain |
| Roger Pujol | Hospital Clínic i Provincial Universitario de Barcelona, Barcelona, Spain |
| Rosa Dosdá | Hospital Clínico Universitario de Valencia, Valencia, Spain |
| Rosa Lardies | Hospital Clínic i Provincial Universitario de Barcelona, Barcelona, Spain |
| Ruben Díaz | Hospital Universitario San Pau, Barcelona, Spain |
| Rubén Villazala | Hospital General Universitario de Ciudad Real, Ciudad Real, Spain |
| Sara Zapatero | Hospital General Universitario Gregorio Marañón, Madrid, Spain |
| Sergio Cabrera | Hospital de Gran Canaria, Dr. Negrín, Gran Canaria, Spain |
| Sergio Sánchez | Hospital General Universitario de Ciudad Real, Ciudad Real, Spain |
| Silvia Martin | Hospital Universitario Río Hortega, Valladolid, Spain |
| Suzana Diaz | Hospital General Universitario Gregorio Marañón, Madrid, Spain |
| Tania Franco | Hospital Universitario Ramón y Cajal, Madrid, Spain |
| Tania Moreno | Hospital Clínico Universitario de Valencia, Valencia, Spain |
| Tania Socorro | Hospital Clínico Universitario de Valencia, Valencia, Spain |
| Vicente Gilabert | Hospital de la Marina Baixa de la Vila Joiosa, Alicante, Spain |
| Victor Balandrón | Hospital General Universitario de Ciudad Real, Ciudad Real, Spain |
| Victoria Moral | Hospital Universitario San Pau, Barcelona, Spain |
| Virgina Cegarra | Hospital Universitario San Pau, Barcelona, Spain |
| Viviana Varón | Hospital Fundación de Alcorcón, Alcorcón, Spain |

*Information and funding*

The iPROVE trial wants to thank all staff who participated, including researchers, nurses, emergency medical technicians, and anyone who has made possible the successful completion of the project. We also thank the technical support provided by the Biomedical Research Foundation Institute. This trial was funded by the Instituto de Salud Carlos III of the Spanish Ministry of Economy and Competitiveness (grant PI14/00829, co-financed by the European Regional Development Fund), and received the support of the Grants Programme of the European Society of Anaesthesiology.

## **THE PROBESE INVESTIGATORS**

*Writing, steering and executive committees*

| Thomas Bluth | University Hospital Carl Gustav Carus, Technische Universität Dresden, Dresden, Germany |
| --- | --- |
| Ary Serpa Neto | Hospital Israelita Albert Einstein, Instituto do Coração, Hospital das Clinicas HCFMUSP, Faculdade de Medicina, Universidade de Sao Paulo São Paulo, Brazil  Amsterdam UMC, University of Amsterdam, Amsterdam, The Netherlands |
| Ilona Bobek | Semmelweis Egyetem, Budapest, Hungary |
| Jaume Canet | Hospital Universitari Germans Trias I Pujol, Barcelona, Spain |
| Gilda Cinnella | Unviersity of Foggia, Foggia, Italy |
| Luc de Baerdemaeker | Ghent University Hospital, Ghent, Belgium |
| Cesare Gregoretti | Città della Salute e dela Scienza, Turin, Italy |
| Göran Hedenstierna | Uppsala University, Uppsala, Sweden |
| Sabrine NT Hemmes | Amsterdam UMC, University of Amsterdam, Amsterdam, The Netherlands |
| Michael Hiesmayr | Medical University Vienna, Vienna, Austria |
| Markus W Hollmann | Amsterdam UMC, University of Amsterdam, Amsterdam, The Netherlands |
| Samir Jaber | Saint Eloi University Hospital, Montpellier, France |
| John Laffey | St. Michael’s Hospital, University of Toronto, Toronto, Canada |
| Marc-Joseph Licker | University Hospitals of Geneva, Geneva, Switzerland |
| Klaus Markstaller | Medical University Vienna, Vienna, Austria |
| Idit Matot, | Tel-Aviv Medical Center, Sackler Medical School, Tel Aviv, Israel |
| Gary H Mills | Sheffield Teaching Hospitals, University of Sheffield, Sheffield, United Kingdom |
| Jan Paul Mulier | AZ Sint Jan Brugge-Oostende AV, Brugge, Belgium |
| Christian Putensen | University Hospital Bonn, Bonn, Germany |
| Rolf Rossaint | Medical Faculty, RWTH Aachen University, Aachen, Germany |
| Jochen Schmitt | University Hospital Carl Gustav Carus, Technische Universität Dresden, Dresden, Germany |
| Mert Senturk | Istanbul University, Istanbul Faculty of Medicine, Istanbul, Turkey |
| Paolo Severgnini | Universita’ dell'Insubria, Azienda Ospedaliera ASST Sette Laghi Ospedale di Circolo e Fondazione Macchi, Varese, Italy |
| Juraj Sprung | Mayo Clinic, Rochester, Minnesota, USA |
| Marcos F Vidal Melo | Massachusetts General Hospital, Boston, Massachusetts, USA |
| Hermann Wrigge | University of Leipzig, Leipzig, Bergmannstrost Hospital Halle, Halle Germany |
| Marcus J Schultz | Hospital Universitario y Politécnico La Fe, Valencia, Amsterdam UMC, University of Amsterdam, Amsterdam, The Netherland  Mahidol University, Bangkok, Thailand |
| Paolo Pelosi | IRCCS San Martino Policlinico Hospital, University of Genoa, Genoa, Italy |
| Marcelo Gama de Abreu | University Hospital Carl Gustav Carus, Technische Universität Dresden, Dresden, Germany |

*Investigators*

| (**, indicates local principal investigators; names are listed in alphabetical order*) | |
| --- | --- |
| Abelha, Fernando* | Centro Hospitalar de São João, Department of Anaesthesiology, Porto, Portugal and Department of Surgery and Physiology, Cardiovascular Research Center, Faculty of Medicine of the University of Porto, Porto, Portugal |
| Abitağaoğlu, Sühayla | Fatih Sultan Mehmet Educational and Research Hospital, İstanbul, Turkey |
| Achilles, Marc* | Marienhospital Wesel, Wesel, Germany |
| Adebesin, Afeez | Imperial College Healthcare NHS Trust, UK |
| Adriaensens, Ine | University Hospital Antwerp, Belgium |
| Ahene, Charles* | Cleveland Clinic Abu Dhabi, Al Maryah Island, Abu Dhabi, UAE |
| Akbar, Fatima | Imperial College Healthcare NHS Trust, UK |
| Al Harbi, Mohammed | Ministry of National Guard Health Affairs, King Abdulaziz Medical City – Riyadh, Anesthesia Department and King Saud Bin Abdulaziz University for Health science, Saudi Arabia |
| Al Khoury al Kallab, Rita | Hôpitaux Universitaires de Genève, Département APSI, service d'anesthésiologie, Switzerland |
| Albanel, Xavier | Hôpitaux Universitaires de Genève, Département APSI, service d'anesthésiologie, Switzerland |
| Aldenkortt, Florence | Hôpitaux Universitaires de Genève, Département APSI, service d'anesthésiologie, Switzerland |
| Alfouzan, Rawan Abdullah Saleh | Ministry of National Guard Health Affairs, King Abdulaziz Medical City – Riyadh, Anesthesia Department, Saudi Arabia |
| Alruqaie, Reef | Ministry of National Guard Health Affairs, King Abdulaziz Medical City – Riyadh, Anesthesia Department, Saudi Arabia |
| Altermatt, Fernando* | Department of Anesthesiology. School of Medicine. Pontificia Universidad Catolica de Chile |
| Araujo, Bruno Luís de Castro* | Department of Anesthesiology, Hospital do Câncer II, National Cancer Institute of Brazil (INCA), Rio de Janeiro, Brazil |
| Arbesú, Genaro | University Hospital Germans Trias i Pujol, ICS, UAB, Spain |
| Artsi, Hanna | Division of Anesthesia, Pain and Critical Care, Tel-Aviv Medical Center affiliated with Sackler Medical School, Tel Aviv University, Tel Aviv, Israel |
| Aurilio, Caterina* | Department of Women, child and General and Specialized Surgery. University of Campania "L. Vanvitelli", Italy. |
| Ayanoglu, Omer Hilmi | Marmara University Pendik Training and Research Hospital, Turkey |
| Bacuzzi, Alessandro | Universita’ dell’Insubria – Dipartimento di Anestesia - Azienda Ospedaliera Asst Settlaghi Ospedale di Circolo e Fondazione Macchi – 21100 Varese - Italy |
| Baig, Harris* | University of Mississippi Medical Center, MS, USA |
| Baird, Yolanda | St Richard’s Hospital, Chichester, UK |
| Balonov, Konstantin* | Department of Anesthesiology and Perioperative Medicine, Tufts Medical Center, Boston, MA, USA |
| Balust, Jaume | Department of Anaesthesiology, Hospital Clínic, Barcelona, Spain. |
| Banks, Samantha | Royal Cornwall Hospital NHS Trust, UK |
| Bao, Xiaodong | Department of Anesthesia, Critical Care and Pain Medicine, Massachusetts General Hospital, Boston, Massachusetts, USA |
| Baumgartner, Mélanie | Hôpitaux Universitaires de Genève, Département APSI, service d'anesthésiologie, Switzerland |
| Belda Tortosa, Isabel* | Hospital Sagrat Cor- Barcelona, Spain |
| Bergamaschi, Alice | Department of Anesthesiology and Intensive Care, Pulmonary Engineering Group, University Hospital Carl Gustav Carus, Technische Universität Dresden, Germany |
| Bergmann, Lars* | Klinik für Anästhesiologie, Intensivmedizin und Schmerztherapie, Universitätsklinikum Knappschaftskrankenhaus Bochum, Bochum, Germany |
| Bigatello, Luca* | St. Elizabeth's Medical Center, MA, USA |
| Biosca Pérez, Elena | Consorcio Hospital General Universitario of Valencia, Spain |
| Birr, Katja | Department of Anesthesiology and Intensive Care, Pulmonary Engineering Group, University Hospital Carl Gustav Carus, Technische Universität Dresden, Germany |
| Bluth, Thomas | Department of Anesthesiology and Intensive Care, Pulmonary Engineering Group, University Hospital Carl Gustav Carus, Technische Universität Dresden, Germany |
| Bojaxhi, Elird | Department of Anesthesiology and Perioperative Medicine, Mayo Clinic Jacksonville, USA |
| Bonenti, Chiara | Universita’ dell’Insubria – Dipartimento di Anestesia - Azienda Ospedaliera Asst Settlaghi Ospedale di Circolo e Fondazione Macchi – 21100 Varese - Italy |
| Bonney, Iwona | Department of Anesthesiology and Perioperative Medicine, Tufts Medical Center, Boston, MA, USA |
| Bos, Elke M.E. | Amsterdam UMC, University of Amsterdam, Department of Anesthesiology, Meibergdreef 9, Amsterdam, The Netherlands |
| Bowman, Sara | Homerton University Hospitals NHS Foundation Trust, UK |
| Braz, Leandro Gobbo | Department of Anesthesiology, Universidade Estadual Paulista, Botucatu/SP, Brazil |
| Brugnoni, Elisa | Universita’ dell’Insubria – Dipartimento di Anestesia - Azienda Ospedaliera Asst Settlaghi Ospedale di Circolo e Fondazione Macchi – 21100 Varese - Italy |
| Brull, Sorin J | Department of Anesthesiology and Perioperative Medicine, College of Medicine, Mayo Clinic Jacksonville, FL, USA |
| Brunetti, Iole | Anestesia e Terapia Intensiva - IRCCS Policlinico San Martino – Genova – Italy |
| Bruni, Andrea | Anesthesia and Intensive Care, Department of Medical and Surgical Sciences, Magna Graeca University, Catanzaro, Italy |
| Buenvenida, Shonie L. | Department of Anesthesiology and Perioperative Medicine, Mayo Clinic, Rochester, Minnesota, USA |
| Busch, Cornelius Johannes* | Department of Anesthesiology, Heidelberg University Hospital, Im Neuenheimer Feld 110, 69120 Heidelberg, Germany |
| Camerini, Giovanni | Dipartimento di Scienze Chirurgiche e Diagnostiche Integrate – DISC – Università degli Studi di Genova – Genova – Italy and Clinica Chirurgica 1 - IRCCS Policlinico San Martino – Genova – Italy |
| Canet, Jaume* | University Hospital Germans Trias i Pujol, ICS, UAB, Spain |
| Capatti, Beatrice | Department Morphology, Surgery and Experimental Medicine. Anesthesia and intensive care universitary section. University of Ferrara. Italy |
| Carmona, Javiera | Department of Anesthesiology. School of Medicine. Pontificia Universidad Catolica de Chile |
| Carungcong, Jaime | Magill Department of Anaesthesia, Chelsea & Westminster NHS Foundation Trust, 369 Fulham Road, London, SW10 9NH, UK |
| Carvalho, Marta | Serviço de Anestesiologia, Centro Hospitalar do Porto, Porto, Portugal |
| Cattan, Anat | Division of Anesthesia, Pain and Critical Care, Tel-Aviv Medical Center affiliated with Sackler Medical School, Tel Aviv University, Tel Aviv, Israel |
| Cavaleiro, Carla | Serviço de Anestesiologia, Centro Hospitalar do Porto, Porto, Portugal and Centro de Investigação Clínica em Anestesiologia, Serviço de Anestesiologia, Centro Hospitalar do Porto, Porto, Portugal |
| Chiumello, Davide* | Dipartimento di Scienze della Salute, Università degli Studi di Milano, Milan, Italy and SC Anestesia e Rianimazione, ASST Santi Paolo e Carlo, Milan, Italy, Centro ricerca cordinata insufficienza respiratoria |
| Ciardo, Stefano | Department Morphology, Surgery and Experimental Medicine. Anesthesia and intensive care universitary section. University of Ferrara. Italy |
| Coburn, Mark | Department of Anesthesiology, Medical Faculty, RWTH Aachen University, Aachen, Germany |
| Colella, Umberto | Department of Women, child and General and Specialized Surgery. University of Campania "L. Vanvitelli", Italy. |
| Contreras, Victor | Department of Anesthesiology. School of Medicine. Pontificia Universidad Catolica de Chile |
| Corman Dincer, Pelin* | Marmara University Pendik Training and Research Hospital, Turkey |
| Cotter, Elizabeth | Section of Critical Care Medicine, Department of Anesthesia and Critical Care, University of Chicago, Chicago, IL, USA |
| Crovetto, Marcia | Department of Anesthesiology. School of Medicine. Pontificia Universidad Catolica de Chile |
| Darrah, William | Department of Anesthesiology, St. Michael’s Hospital, University of Toronto, Toronto, ON, Canada |
| Davies, Simon* | York Teaching Hospitals NHS Foundation Trust, UK |
| de Baerdemaeker, Luc* | Ghent University Hospital, Ghent, Belgium. Corneel Heymanslaan 10, 9000 Gent, Belgium |
| De Hert, Stefan | Ghent University Hospital, Ghent, Belgium. Corneel Heymanslaan 10, 9000 Gent, Belgium |
| Del Cojo Peces, Enrique | Área de Salud Don Benito-Villanueva/Don Benito-Villanueva Health Area, Spain. |
| Delphin, Ellise | Montefiore Medical Center, Bronx, NY 10467, USA |
| Diaper, John | Hôpitaux Universitaires de Genève, Département APSI, service d'anesthésiologie, Switzerland |
| do Nascimento Junior, Paulo* | Department of Anesthesiology, Universidade Estadual Paulista, Botucatu/SP, Brazil |
| Donatiello, Valerio | Department of Women, child and General and Specialized Surgery. University of Campania "L. Vanvitelli", Italy. |
| Dong, Jing | Department of Anesthesiology, Fudan University Shanghai Cancer Center, Shanghai, China. Department of Oncology, Shanghai Medical College, Fudan University, Shanghai, China |
| Dourado, Maria do Socorro | Associação Hospitalar Beneficente São Vicente de Paulo, Brazil |
| Dullenkopf, Alexander* | Frauenfeld Cantonal Hospital, Switzerland |
| Ebner, Felix | Klinik für Anästhesiologie, Intensivmedizin und Schmerztherapie,Universitätsklinikum Knappschaftskrankenhaus Bochum, Bochum, Germany |
| Elgendy, Hamed* | Departments of Anesthesiology, King Abdullah Medical City, Makkah, Saudi Arabia and Assiut University, Egypt and Hamad Medical Corporation, Qatar. |
| Ellenberger, Christoph | Hôpitaux Universitaires de Genève, Département APSI, service d'anesthésiologie, Switzerland |
| Erdoğan Arı, Dilek* | Fatih Sultan Mehmet Educational and Research Hospital, İstanbul, Turkey |
| Ermert, Thomas | Department of Anesthesiology, Intensive Care and Pain Medicine, University Hospital Münster, Münster, Germany |
| Farah, Fadi | St. Elizabeth's Medical Center, MA, USA |
| Fernandez-Bustamante, Ana* | University of Colorado School of Medicine, Department of Anesthesiology, CO, USA |
| Ferreira, Cristina | Serviço de Anestesiologia, Centro Hospitalar do Porto, Porto, Portugal |
| Fiore, Marco | Department of Women, child and General and Specialized Surgery. University of Campania "L. Vanvitelli", Italy. |
| Fonte, Ana | Department of Anesthesiology from Centro Hospitalar de Entre o Douro e Vouga, EPE – São Sebastião Hospital, Santa Maria da Feira, Portugal |
| Fortià Palahí, Christina | Hospital Sagrat Cor- Barcelona, Spain |
| Galimberti, Andrea | Dipartimento di Scienze della Salute, Università degli Studi di Milano, Milan, Italy and SC Anestesia e Rianimazione, Asst Santi Paolo e Carlo, Milan, Italy, Centro ricerca cordinata insufficienza respiratoria |
| Gama de Abreu, Marcelo* | Department of Anesthesiology and Intensive Care, Pulmonary Engineering Group, University Hospital Carl Gustav Carus, Technische Universität Dresden, Germany |
| Garofano, Najia | Hôpitaux Universitaires de Genève, Département APSI, service d'anesthésiologie, Switzerland |
| Giaccari, Luca Gregorio | Department of Women, child and General and Specialized Surgery. University of Campania "L. Vanvitelli", Italy. |
| Gilsanz, Fernando | Department of Anesthesiology and Surgical Critical Care, Hospital Universitario La Paz, Madrid, Spain |
| Girrbach, Felix | Department of Anaesthesiology and Intensive Care Medicine, University of Leipzig, Leipzig, Germany; |
| Gobbi, Luca | Department Morphology, Surgery and Experimental Medicine. Anesthesia and intensive care universitary section. University of Ferrara. Italy |
| Godfried,Marc Bernard* | Department of Anesthesiology, Onze Lieve Vrouwe Gasthuis, Amsterdam, The Netherlands |
| Goettel, Nicolai* | Department of Anesthesia, Surgical Intensive Care, Prehospital Emergency Medicine and Pain Therapy, University Hospital Basel, University of Basel, Basel, Switzerland |
| Goldstein, Peter A.* | Weill Cornell Medicine, New York, NY, USA |
| Goren, Or | Division of Anesthesia, Pain and Critical Care, Tel-Aviv Medical Center affiliated with Sackler Medical School, Tel Aviv University, Tel Aviv, Israel |
| Gorlin, Andrew | Mayo Clinic Arizona, 5777 East Mayo Blvd, Phoenix, AZ, 85054, USA |
| Granell Gil, Manuel* | Consorcio Hospital General Universitario of Valencia, Spain |
| Gratarola, Angelo | Anestesia e Rianimazione - IRCCS Policlinico San Martino – Genova – Italy |
| Graterol, Juan* | Royal Cornwall Hospital NHS Trust, UK |
| Guyon, Pierre | Hôpitaux Universitaires de Genève, Département APSI, service d'anesthésiologie, Switzerland |
| Haire, Kevin | Magill Department of Anaesthesia, Chelsea & Westminster NHS Foundation Trust, 369 Fulham Road, London, SW10 9NH, UK |
| Harou, Philippe | Polyclinique Montier la Celle, France |
| Helf, Antonia | Department of Anaesthesiology, University Hospital Würzburg, Würzburg, Germany |
| Hemmes, Sabrine N. T.* | Amsterdam UMC, University of Amsterdam, Department of Anesthesiology, Meibergdreef 9, Amsterdam, The Netherlands |
| Hempel, Gunther | Department of Anaesthesiology and Intensive Care Medicine, University of Leipzig, Leipzig, Germany; |
| Hernández Cádiz, María José | Consorcio Hospital General Universitario of Valencia, Spain |
| Heyse, Björn | Ghent University Hospital, Ghent, Belgium. Corneel Heymanslaan 10, 9000 Gent, Belgium |
| Hollmann, Markus W.* | Amsterdam UMC, University of Amsterdam, Department of Anesthesiology, Meibergdreef 9, Amsterdam, The Netherlands |
| Huercio, Ivan | Department of Anesthesiology and Surgical Critical Care, Hospital Universitario La Paz, Madrid, Spain |
| Ilievska, Jasmina | University Clinic of Surgery “Ss. Naum Ohridski”, Faculty of Medicine, University “Ss. Cyril and Methodius”, Skopje, Macedonia |
| Jakus, Lien | Hôpitaux Universitaires de Genève, Département APSI, service d'anesthésiologie, Switzerland |
| Jeganath, Vijay | Royal Stoke University Hospital, UHNM NHS Trust, Stoke-on-Trent, Staffordshire, ST4 6QG, UK |
| Jelting, Yvonne | Department of Anaesthesiology, University Hospital Würzburg, Würzburg, Germany |
| Jung, Minoa | Hôpitaux Universitaires de Genève, Département APSI, service d'anesthésiologie, Switzerland |
| Kabon, Barbara* | Department of Anaesthesia,Critical Care and Pain Medicine, Medical University Vienna, Austria |
| Kacha, Aalok* | Section of Critical Care Medicine, Department of Anesthesia and Critical Care, University of Chicago, Chicago, IL, USA |
| Karaman Ilić, Maja | Clinical hospital Sveti Duh, JJ Strossmayer, Faculty of Medicine, Zagreb, Croatia |
| Karuppiah, Arunthevaraja | St. Elizabeth's Medical Center, MA, USA |
| Kavas, Ayse Duygu | Marmara University Pendik Training and Research Hospital, Turkey |
| Keli Barcelos, Gleicy | Hôpitaux Universitaires de Genève, Département APSI, service d'anesthésiologie, Switzerland |
| Kellogg, Todd A. | Department of Surgery, Mayo Clinic, Rochester, Minnesota, USA |
| Kemper, Johann | Department of Anesthesiology, University Hospital Duesseldorf, Heinrich-Heine University Duesseldorf, Duesseldorf, Germany |
| Kerbrat, Romain | Hôpitaux Universitaires de Genève, Département APSI, service d'anesthésiologie, Switzerland |
| Khodr, Suraya | Cleveland Clinic Abu Dhabi, Al Maryah Island, Abu Dhabi, UAE |
| Kienbaum, Peter | Department of Anesthesiology, University Hospital Duesseldorf, Heinrich-Heine University Duesseldorf, Duesseldorf, Germany |
| Kir, Bunyamin | Marmara University Pendik Training and Research Hospital, Turkey |
| Kiss, Thomas | Department of Anesthesiology and Intensive Care, Pulmonary Engineering Group, University Hospital Carl Gustav Carus, Technische Universität Dresden, Germany |
| Kivrak, Selin | Hôpitaux Universitaires de Genève, Département APSI, service d'anesthésiologie, Switzerland |
| Klarić, Vlasta* | Clinic of anesthesiology, reanimatology and intensive care medicine, University Hospital Dubrava, Zagreb, Croatia |
| Koch, Thea | Department of Anesthesiology and Intensive Care, Pulmonary Engineering Group, University Hospital Carl Gustav Carus, Technische Universität Dresden, Germany |
| Köksal, Ceren | Fatih Sultan Mehmet Educational and Research Hospital, İstanbul, Turkey |
| Kowark, Ana | Department of Anesthesiology, Medical Faculty, RWTH Aachen University, Aachen, Germany |
| Kranke, Peter* | Department of Anaesthesiology, University Hospital Würzburg, Würzburg, Germany |
| Kuvaki, Bahar* | Dokuz Eylül University Faculty of Medicine, Department of Anaesthesiology and Intensive Care, Turkey |
| Kuzmanovska, Biljana | University clinic for Traumatology, Orthopedics, Anesthesia, Reanimation, Intensive care and Emmergency center - Skopje, Department of Anesthesiology, Reanimation and Intensive care Medicine - Skopje, Macedonia |
| Laffey, John* | Department of Anesthesiology, St. Michael’s Hospital, University of Toronto, Toronto, ON, Canada |
| Lange, Mirko | Department of Anaesthesiology and Intensive Care Medicine, University of Leipzig, Leipzig, Germany; |
| Lemos, Marília Freitas de | Department of Anesthesiology, Hospital do Câncer II, National Cancer Institute of Brazil (INCA), Rio de Janeiro, Brazil |
| Licker, Marc-Joseph* | Hôpitaux Universitaires de Genève, Département APSI, service d'anesthésiologie; Faculty of Medicine, University of Geneva, Switzerland |
| López-Baamonde, Manuel | Department of Anaesthesiology, Hospital Clínic, Barcelona, Spain. |
| López-Hernández, Antonio | Department of Anaesthesiology, Hospital Clínic, Barcelona, Spain. |
| Lopez-Martinez, Mercedes | Department of Anesthesiology and Surgical Critical Care, Hospital Universitario La Paz, Madrid, Spain |
| Luise, Stéphane | Hôpitaux Universitaires de Genève, Département APSI, service d'anesthésiologie, Switzerland |
| MacGregor, Mark* | Ashford and St Peters NHS Foundation Trust, UK |
| Magalhães, Danielle* | Associação Hospitalar Beneficente São Vicente de Paulo, Brazil |
| Maillard, Julien | Hôpitaux Universitaires de Genève, Département APSI, service d'anesthésiologie, Switzerland |
| Malerbi, Patrizia | Anestesia e Rianimazione - IRCCS Policlinico San Martino – Genova – Italy |
| Manimekalai, Natesan | University of Mississippi Medical Center, MS, USA |
| Margarson, Michael* | St Richard’s Hospital, Chichester, UK |
| Markstaller, Klaus* | Department of Anaesthesia,Critical Care and Pain Medicine, Medical University Vienna, Austria |
| Martin, Archer K | Department of Anesthesiology and Perioperative Medicine, College of Medicine, Mayo Clinic Jacksonville, FL, USA |
| Martin, David P. | Department of Anesthesiology and Perioperative Medicine, Mayo Clinic, Rochester, Minnesota, USA |
| Martin, Yvette N. | Department of Anesthesiology and Perioperative Medicine, Mayo Clinic, Rochester, Minnesota, USA |
| Martínez-Ocon, Julia | Department of Anaesthesiology, Hospital Clínic, Barcelona, Spain. |
| Martin-Loeches, Ignacio* | Department of Intensive Care Medicine, Multidisciplinary Intensive Care Research Organization (MICRO), St. James's Hospital, Dublin, Ireland |
| Maseda, Emilio | Department of Anesthesiology and Surgical Critical Care, Hospital Universitario La Paz, Madrid, Spain |
| Matot, Idit* | Division of Anesthesia, Pain and Critical Care, Tel-Aviv Medical Center affiliated with Sackler Medical School, Tel Aviv University, Tel Aviv, Israel |
| McAuliffe, Niamh | Department of Anesthesiology, St. Michael’s Hospital, University of Toronto, Toronto, ON, Canada |
| McKenzie, Travis J. | Department of Surgery, Mayo Clinic, Rochester, Minnesota, USA |
| Medina, Paulina | Department of Anesthesiology. School of Medicine. Pontificia Universidad Catolica de Chile |
| Meersch, Melanie | Department of Anesthesiology, Intensive Care and Pain Medicine, University Hospital Münster, Münster, Germany |
| Menzen, Angelika* | St. Marienhospital gGmbh Friesoythe, Friesoythe, Germany |
| Mertens, Els* | University Hospital Antwerp, Belgium |
| Meurer, Bernd | Marienhospital Wesel, Wesel, Germany |
| Meyer-Treschan, Tanja* | Department of Anesthesiology, University Hospital Duesseldorf, Heinrich-Heine University Duesseldorf, Duesseldorf, Germany |
| Miao, Changhong* | Department of Anesthesiology, Fudan University Shanghai Cancer Center, Shanghai, China. Department of Oncology, Shanghai Medical College, Fudan University, Shanghai, China |
| Micalizzi, Camilla | Dipartimento di Scienze Chirurgiche e Diagnostiche Integrate – DISC – Università degli Studi di Genova – Genova – Italy and Anestesia e Terapia Intensiva - IRCCS Policlinico San Martino – Genova – Italy |
| Milić, Morena | Clinic of anesthesiology, reanimatology and intensive care medicine, University Hospital Dubrava, Zagreb, Croatia |
| Módolo, Norma Sueli Pinheiro | Department of Anesthesiology, Universidade Estadual Paulista, Botucatu/SP, Brazil |
| Moine, Pierre | University of Colorado School of Medicine, Department of Anesthesiology, CO, USA |
| Mölders, Patrick | Klinik für Anästhesiologie, Intensivmedizin und Schmerztherapie,Universitätsklinikum Knappschaftskrankenhaus Bochum, Bochum, Germany |
| Montero-Feijoo, Ana | Department of Anesthesiology and Surgical Critical Care, Hospital Universitario La Paz, Madrid, Spain |
| Moret, Enrique | University Hospital Germans Trias i Pujol, ICS, UAB, Spain |
| Muller, Markus K. | Frauenfeld Cantonal Hospital, Switzerland |
| Murphy, Zoe | York Teaching Hospitals NHS Foundation Trust, UK |
| Nalwaya, Pramod* | Royal Stoke University Hospital, UHNM NHS Trust, Stoke-on-Trent, Staffordshire, ST4 6QG, UK |
| Naumovski, Filip* | University clinic for Traumatology, Orthopedics, Anesthesia, Reanimation, Intensive care and Emmergency center - Skopje, Department of Anesthesiology, Reanimation and Intensive care Medicine - Skopje, Macedonia |
| Navalesi, Paolo* | Anesthesia and Intensive Care, Department of Medical and Surgical Sciences, Magna Graeca University, Catanzaro, Italy |
| Navarro e Lima, Lais Helena | Department of Anesthesiology, Universidade Estadual Paulista, Botucatu/SP, Brazil |
| Nesek Adam, Višnja* | Clinical hospital Sveti Duh, JJ Strossmayer, Faculty of Medicine, Zagreb, Croatia |
| Neumann, Claudia | Department of Anaesthesiology and Intensive Care Medicine, University Hospital of Bonn, Bonn, Germany |
| Newell, Christopher* | Southmead Hospital, North Bristol NHS Trust, UK |
| Nisnevitch, Zoulfira* | Montefiore Medical Center, Bronx, NY 10467, USA |
| Nizamuddin, Junaid | Section of Critical Care Medicine, Department of Anesthesia and Critical Care, University of Chicago, Chicago, IL, USA |
| Novazzi, Cecilia | Universita’ dell’Insubria – Dipartimento di Anestesia - Azienda Ospedaliera Asst Settlaghi Ospedale di Circolo e Fondazione Macchi – 21100 Varese - Italy |
| O'Connor, Michael | Section of Critical Care Medicine, Department of Anesthesia and Critical Care, University of Chicago, Chicago, IL, USA |
| Oprea, Günther | Klinik für Anästhesiologie, Intensivmedizin und Schmerztherapie, Universitätsklinikum Knappschaftskrankenhaus Bochum, Bochum, Germany |
| Orhan Sungur, Mukadder | Istanbul University, Istanbul Faculty of Medicine, Turkey |
| Özbilgin, Şule | Dokuz Eylül University Faculty of Medicine, Department of Anaesthesiology and Intensive Care, Turkey |
| Pace, Maria Caterina | Department of Women, child and General and Specialized Surgery. University of Campania "L. Vanvitelli", Italy. |
| Pacheco, Marcos | Department of Anesthesiology from Centro Hospitalar de Entre o Douro e Vouga, EPE – São Sebastião Hospital, Santa Maria da Feira, Portugal |
| Packianathaswamy, Balaji* | Hull and East Yorkshire Hospitals NHS Trust, UK |
| Palma Gonzalez, Estefania | Área de Salud Don Benito-Villanueva/Don Benito-Villanueva Health Area, Spain. |
| Papaspyros, Fotios* | POLYCLINIQUE MONTIER LA CELLE, France |
| Paredes, Sebastián | Department of Anesthesiology. School of Medicine. Pontificia Universidad Catolica de Chile |
| Passavanti, Maria Beatrice | Department of Women, child and General and Specialized Surgery. University of Campania "L. Vanvitelli", Italy. |
| Pedemonte, Juan Cristobal | Department of Anesthesiology. School of Medicine. Pontificia Universidad Catolica de Chile |
| Pelosi, Paolo* | Dipartimento di Scienze Chirurgiche e Diagnostiche Integrate – DISC – Università degli Studi di Genova – Genova – Italy and Anestesia e Terapia Intensiva - IRCCS Policlinico San Martino – Genova – Italy |
| Peremin, Sanja | Clinic of anesthesiology, reanimatology and intensive care medicine, University Hospital Dubrava, Zagreb, Croatia |
| Philipsenburg, Christoph | Department of Anesthesiology, Heidelberg University Hospital, Im Neuenheimer Feld 110, 69120 Heidelberg, Germany |
| Pinho, Daniela | Serviço de Anestesiologia, Centro Hospitalar do Porto, Porto, Portugal |
| Pinho, Silvia | Serviço de Anestesiologia, Centro Hospitalar do Porto, Porto, Portugal |
| Posthuma, Linda M. | Amsterdam UMC, University of Amsterdam, Department of Anesthesiology, Meibergdreef 9, Amsterdam, The Netherlands |
| Pota, Vincenzo | Department of Women, child and General and Specialized Surgery. University of Campania "L. Vanvitelli", Italy. |
| Preckel, Benedikt | Amsterdam UMC, University of Amsterdam, Department of Anesthesiology, Meibergdreef 9, Amsterdam, The Netherlands |
| Priani, Paolo | Department Morphology, Surgery and Experimental Medicine. Anesthesia and intensive care universitary section. University of Ferrara. Italy |
| Putensen, Christian* | Department of Anaesthesiology and Intensive Care Medicine, University Hospital of Bonn, Bonn, Germany |
| Rached, Mohamed Aymen | Hôpitaux Universitaires de Genève, Département APSI, service d'anesthésiologie, Switzerland |
| Radoeshki, Aleksandar | University Clinic of Surgery “Ss. Naum Ohridski”, Faculty of Medicine, University “Ss. Cyril and Methodius”, Skopje, Macedonia |
| Ragazzi, Riccardo | Department Morphology, Surgery and Experimental Medicine. Anesthesia and intensive care universitary section. University of Ferrara. Italy |
| Rajamanickam, Tamilselvan | Royal Stoke University Hospital, UHNM NHS Trust, Stoke-on-Trent, Staffordshire, ST4 6QG, UK |
| Rajamohan, Arthi | Department of Anesthesiology, St. Michael’s Hospital, University of Toronto, Toronto, ON, Canada |
| Ramakrishna, Harish* | Mayo Clinic Arizona, 5777 East Mayo Blvd, Phoenix, AZ, 85054, USA |
| Rangarajan, Desikan* | Homerton University Hospitals NHS Foundation Trust, UK |
| Reiterer, Christian | Department of Anaesthesia,Critical Care and Pain Medicine, Medical University Vienna, Austria |
| Renew, J. Ross | Department of Anesthesiology and Perioperative Medicine, Mayo Clinic Jacksonville, USA |
| Reynaud, Thomas | Hôpitaux Universitaires de Genève, Département APSI, service d'anesthésiologie, Switzerland |
| Rhys, Rhidian | Southmead Hospital, North Bristol NHS Trust, UK |
| Rivas, Eva* | Department of Anaesthesiology, Hospital Clínic, IDIBAPS, Universitat de Barcelona, Spain |
| Robitzky, Luisa | Klinik für Anästhesiologie, Intensivmedizin und Schmerztherapie, Universitätsklinikum Knappschaftskrankenhaus Bochum, Bochum, Germany |
| Rossaint, Rolf* | Department of Anesthesiology, Medical Faculty, RWTH Aachen University, Aachen, Germany |
| Rubulotta, Francesca* | Imperial College Healthcare NHS Trust, UK |
| S. Machado, Humberto* | Serviço de Anestesiologia, Centro Hospitalar do Porto, Porto, Portugal and Instituto Ciências Biomédicas Abel Salazar, Universidade do Porto, Porto, Portugal and Centro de Investigação Clínica em Anestesiologia, Serviço de Anestesiologia, Centro Hospitalar do Porto, Porto, Portugal |
| S. Nunes, Catarina | Universidade Aberta, Departamento de Ciências e Tecnologia, Porto, Portugal and Centro de Investigação Clínica em Anestesiologia, Serviço de Anestesiologia, Centro Hospitalar do Porto, Porto, Portugal |
| Sabbatini, Giovanni | Dipartimento di Scienze della Salute, Università degli Studi di Milano, Milan, Italy and SC Anestesia e Rianimazione, ASST Santi Paolo e Carlo, Milan, Italy, Centro ricerca cordinata insufficienza respiratoria |
| Samuels, Jon D | Weill Cornell Medicine, Department of Anesthesiology, New York-Presbyterian Hospital, New York, NY, USA |
| Sanahuja, Josep Martí | Department of Anaesthesiology, Hospital Clínic, Barcelona, Spain. |
| Sansone, Pasquale | Department of Women, child and General and Specialized Surgery. University of Campania "L. Vanvitelli", Italy. |
| Santos, Alice | Department of Anaesthesiology, Centro Hospitalar São João, Porto, Portugal |
| Sayedalahl, Mohamed | Departments of Anesthesiology, King Abdullah Medical City, Makkah, Saudi Arabia and Mansoura University, Egypt |
| Schaefer, Maximilian S. | Department of Anesthesiology, University Hospital Duesseldorf, Heinrich-Heine University Duesseldorf, Duesseldorf, Germany |
| Scharffenberg, Martin | Department of Anesthesiology and Intensive Care, Pulmonary Engineering Group, University Hospital Carl Gustav Carus, Technische Universität Dresden, Germany |
| Schiffer, Eduardo | Hôpitaux Universitaires de Genève, Département APSI, service d'anesthésiologie; Faculty of Medicine, University of Geneva, Switzerland |
| Schliewe, Nadja | Department of Anaesthesiology and Intensive Care Medicine, University of Leipzig, Leipzig, Germany; |
| Schorer, Raoul | Hôpitaux Universitaires de Genève, Département APSI, service d'anesthésiologie, Switzerland |
| Schultz, Marcus J. | Amsterdam UMC, University of Amsterdam, Department of Anesthesiology, Meibergdreef 9, Amsterdam, The Netherlands |
| Schumann, Roman | Department of Anesthesiology and Perioperative Medicine, Tufts Medical Center, Boston, MA, USA |
| Selmo, Gabriele | Universita’ dell’Insubria – Dipartimento di Anestesia - Azienda Ospedaliera Asst Settlaghi Ospedale di Circolo e Fondazione Macchi – 21100 Varese - Italy |
| Sendra, Mar | University Hospital Germans Trias i Pujol, ICS, UAB, Spain |
| Senturk, Mert* | Istanbul University, Istanbul Faculty of Medicine, Turkey |
| Severgnini, Paolo* | Universita’ dell’Insubria – Dipartimento di Anestesia - Azienda Ospedaliera Asst Settlaghi Ospedale di Circolo e Fondazione Macchi – 21100 Varese - Italy |
| Shaw, Kate | York Teaching Hospitals NHS Foundation Trust, UK |
| Shosholcheva, Mirjana* | University Clinic of Surgery “Ss. Naum Ohridski”, Faculty of Medicine, University “Ss. Cyril and Methodius”, Skopje, Macedonia |
| Sibai, Abdulrazak* | MINISTRY OF NATIONAL GUARD HEALTH AFFAIRS, KING ABDULAZIZ MEDICAL CITY – RIYADH, Anesthesia Department, Saudi Arabia |
| Simon, Philipp | Department of Anaesthesiology and Intensive Care Medicine, University of Leipzig, Leipzig, Germany; |
| Simonassi, Francesca | Dipartimento di Scienze Chirurgiche e Diagnostiche Integrate – DISC – Università degli Studi di Genova – Genova – Italy and Anestesia e Terapia Intensiva - IRCCS Policlinico San Martino – Genova – Italy |
| Sinno, Claudia | Universita’ dell’Insubria – Dipartimento di Anestesia - Azienda Ospedaliera Asst Settlaghi Ospedale di Circolo e Fondazione Macchi – 21100 Varese - Italy |
| Sivrikoz, Nukhet | Istanbul University, Istanbul Faculty of Medicine, Turkey |
| Skandalou, Vasiliki* | Alexandra General Hospital, Athens, Greece |
| Smith, Neil | Hull and East Yorkshire Hospitals NHS Trust, UK |
| Soares, Maria | Serviço de Anestesiologia, Centro Hospitalar do Porto, Porto, Portugal |
| Socorro Artiles, Tania | University Clinic Hospital Valencia, Spain |
| Sousa Castro, Diogo* | Department of Anesthesiology from Centro Hospitalar de Entre o Douro e Vouga, EPE – São Sebastião Hospital, Santa Maria da Feira, Portugal |
| Sousa, Miguel | Department of Anesthesiology from Centro Hospitalar de Entre o Douro e Vouga, EPE – São Sebastião Hospital, Santa Maria da Feira, Portugal |
| Spadaro, Savino* | Department Morphology, Surgery and Experimental Medicine. Anesthesia and intensive care universitary section. University of Ferrara. Italy |
| Sprung, Juraj* | Department of Anesthesiology and Perioperative Medicine, Mayo Clinic, Rochester, Minnesota, USA |
| Stamatakis, Emmanouil | Alexandra General Hospital, Athens, Greece |
| Steiner, Luzius A. | Department of Anesthesia, Surgical Intensive Care, Prehospital Emergency Medicine and Pain Therapy, University Hospital Basel, University of Basel, Basel, Switzerland |
| Stevenazzi, Andrea | University Hospital Germans Trias i Pujol, ICS, UAB, Spain |
| Suarez-de-la-Rica, Alejandro* | Department of Anesthesiology and Surgical Critical Care, Hospital Universitario La Paz, Madrid, Spain |
| Suppan, Mélanie | Hôpitaux Universitaires de Genève, Département APSI, service d'anesthésiologie, Switzerland |
| Teichmann, Robert | Department of Anesthesiology and Intensive Care, Pulmonary Engineering Group, University Hospital Carl Gustav Carus, Technische Universität Dresden, Germany |
| Tena Guerrero, José Maria* | Área de Salud Don Benito-Villanueva/Don Benito-Villanueva Health Area, Spain. |
| Thiel, Bram | Department of Anesthesiology, Onze Lieve Vrouwe Gasthuis, Amsterdam, The Netherlands |
| Tolós, Raquel | University Hospital Germans Trias i Pujol, ICS, UAB, Spain |
| Tore Altun, Gulbin | Marmara University Pendik Training and Research Hospital, Turkey |
| Tucci, Michelle | University of Mississippi Medical Center, MS, USA |
| Turnbull, Zachary A. | Weill Cornell Medicine, New York, NY, USA |
| Turudić, Žana | Clinic of anesthesiology, reanimatology and intensive care medicine, University Hospital Dubrava, Zagreb, Croatia |
| Unterberg, Matthias | Klinik für Anästhesiologie, Intensivmedizin und Schmerztherapie, Universitätsklinikum Knappschaftskrankenhaus Bochum, Bochum, Germany |
| Van Limmen, Jurgen | Ghent University Hospital, Ghent, Belgium. Corneel Heymanslaan 10, 9000 Gent, Belgium |
| Van Nieuwenhove, Yves | Ghent University Hospital, Ghent, Belgium. Corneel Heymanslaan 10, 9000 Gent, Belgium |
| Van Waesberghe, Julia | Department of Anesthesiology, Medical Faculty, RWTH Aachen University, Aachen, Germany |
| Vidal Melo, Marcos Francisco* | Department of Anesthesia, Critical Care and Pain Medicine, Massachusetts General Hospital, Boston, Massachusetts, USA |
| Vitković, Bibiana | Clinic of anesthesiology, reanimatology and intensive care medicine, University Hospital Dubrava, Zagreb, Croatia |
| Vivona, Luigi | Department of Anesthesiology and Intensive Care, Pulmonary Engineering Group, University Hospital Carl Gustav Carus, Technische Universität Dresden, Germany |
| Vizcaychipi, Marcela* | Magill Department of Anaesthesia, Chelsea & Westminster NHS Foundation Trust, 369 Fulham Road, London, SW10 9NH, UK |
| Volta, Carlo Alberto | Department Morphology, Surgery and Experimental Medicine. Anesthesia and intensive care universitary section. University of Ferrara. Italy |
| Weber, Anne | Hôpitaux Universitaires de Genève, Département APSI, service d'anesthésiologie, Switzerland |
| Weingarten, Toby N. | Department of Anesthesiology and Perioperative Medicine, Mayo Clinic, Rochester, Minnesota, USA |
| Wittenstein, Jakob | Department of Anesthesiology and Intensive Care, Pulmonary Engineering Group, University Hospital Carl Gustav Carus, Technische Universität Dresden, Germany |
| Wrigge, Hermann* | Department of Anaesthesiology and Intensive Care Medicine, University of Leipzig, Leipzig, Germany and Department of Anaesthesiology, Intensive Care and Emergency Medicine, Pain Therapy; Bergmannstrost Hospital Halle, Halle, Germany |
| Wyffels, Piet | Ghent University Hospital, Ghent, Belgium. Corneel Heymanslaan 10, 9000 Gent, Belgium |
| Yagüe, Julio | Department of Anesthesiology and Surgical Critical Care, Hospital Universitario La Paz, Madrid, Spain |
| Yates, David | York Teaching Hospitals NHS Foundation Trust, UK |
| Yavru, Ayşen | Istanbul University, Istanbul Faculty of Medicine, Turkey |
| Zac, Lilach | Division of Anesthesia, Pain and Critical Care, Tel-Aviv Medical Center affiliated with Sackler Medical School, Tel Aviv University, Tel Aviv, Israel |
| Zhong, Jing | Department of Anesthesiology, Fudan University Shanghai Cancer Center, Shanghai, China. Department of Oncology, Shanghai Medical College, Fudan University, Shanghai, China |

*Information and funding*

The Clinical Trials Network of the European Society of Anaesthesiology provided financial support for the steering committee meetings, onsite visits to participating sites, for the building of the electronic data capture system, and for the advertising of the study. The Technische Universität Dresden provided logistical support for the coordinating site. The Conselho Nacional de Desenvolvimento Científico e Tecnológico provided financial support for insurance in Brazil. The Association of Anaesthetists of Great Britain and Ireland and the Northern Ireland Society of Anaesthetists provided financial support for the participating sites in the United Kingdom.

# PRISMA-IPD Checklist of items to include when reporting a systematic review and meta-analysis of individual participant data (IPD)

| **PRISMA-IPD**  **Section/topic** | **Item No** | **Checklist item** | **Reported on page** |
| --- | --- | --- | --- |
| **Title** | | | |
| Title | 1 | Identify the report as a systematic review and meta-analysis of individual participant data. | Title |
| **Abstract** | | | |
| Structured summary | 2 | Provide a structured summary including as applicable: | Abstract  Page 4,5 |
|  |  | **Background**: state research question and main objectives, with information on participants, interventions, comparators and outcomes. |  |
|  |  | **Methods**: report eligibility criteria; data sources including dates of last bibliographic search or elicitation, noting that IPD were sought; methods of assessing risk of bias. |  |
|  |  | **Results**: provide number and type of studies and participants identified and number (%) obtained; summary effect estimates for main outcomes (benefits and harms) with confidence intervals and measures of statistical heterogeneity. Describe the direction and size of summary effects in terms meaningful to those who would put findings into practice. |  |
|  |  | **Discussion:** state main strengths and limitations of the evidence, general interpretation of the results and any important implications. |  |
|  |  | **Other:** report primary funding source, registration number and registry name for the systematic review and IPD meta-analysis. |  |
| **Introduction** | | | |
| Rationale | 3 | Describe the rationale for the review in the context of what is already known. | 6 |
| Objectives | 4 | Provide an explicit statement of the questions being addressed with reference, as applicable, to participants, interventions, comparisons, outcomes and study design (PICOS). Include any hypotheses that relate to particular types of participant-level subgroups. | 6 |
| **Methods** | | | |
| Protocol and registration | 5 | Indicate if a protocol exists and where it can be accessed. If available, provide registration information including registration number and registry name. Provide publication details, if applicable. | 7 |
| Eligibility criteria | 6 | Specify inclusion and exclusion criteria including those relating to participants, interventions, comparisons, outcomes, study design and characteristics (e.g. years when conducted, required minimum follow-up). Note whether these were applied at the study or individual level i.e. whether eligible participants were included (and ineligible participants excluded) from a study that included a wider population than specified by the review inclusion criteria. The rationale for criteria should be stated. | 8 |
| Identifying studies - information sources | 7 | Describe all methods of identifying published and unpublished studies including, as applicable: which bibliographic databases were searched with dates of coverage; details of any hand searching including of conference proceedings; use of study registers and agency or company databases; contact with the original research team and experts in the field; open adverts and surveys. Give the date of last search or elicitation. | 7 |
| Identifying studies - search | 8 | Present the full electronic search strategy for at least one database, including any limits used, such that it could be repeated. | 7 |
| Study selection processes | 9 | State the process for determining which studies were eligible for inclusion. | - |
| Data collection processes | 10 | Describe how IPD were requested, collected and managed, including any processes for querying and confirming data with investigators. If IPD were not sought from any eligible study, the reason for this should be stated (for each such study). | 7 |
|  |  | If applicable, describe how any studies for which IPD were not available were dealt with. This should include whether, how and what aggregate data were sought or extracted from study reports and publications (such as extracting data independently in duplicate) and any processes for obtaining and confirming these data with investigators. |  |
| Data items | 11 | Describe how the information and variables to be collected were chosen. List and define all study level and participant level data that were sought, including baseline and follow-up information. If applicable, describe methods of standardising or translating variables within the IPD datasets to ensure common scales or measurements across studies. | 8,9 |
| IPD integrity | A1 | Describe what aspects of IPD were subject to data checking (such as sequence generation, data consistency and completeness, baseline imbalance) and how this was done. | 8 |
| Risk of bias assessment in individual studies. | 12 | Describe methods used to assess risk of bias in the individual studies and whether this was applied separately for each outcome. If applicable, describe how findings of IPD checking were used to inform the assessment. Report if and how risk of bias assessment was used in any data synthesis. | **-** |
| Specification of outcomes and effect measures | 13 | State all treatment comparisons of interests. State all outcomes addressed and define them in detail. State whether they were pre-specified for the review and, if applicable, whether they were primary/main or secondary/additional outcomes. Give the principal measures of effect (such as risk ratio, hazard ratio, difference in means) used for each outcome. | 9,10 |
| Synthesis methods | 14 | Describe the meta-analysis methods used to synthesise IPD. Specify any statistical methods and models used. Issues should include (but are not restricted to):   - Use of a one-stage or two-stage approach. - How effect estimates were generated separately within each study and combined across studies (where applicable). - Specification of one-stage models (where applicable) including how clustering of patients within studies was accounted for. - Use of fixed or random effects models and any other model assumptions, such as proportional hazards. - How (summary) survival curves were generated (where applicable). - Methods for quantifying statistical heterogeneity (such as I^2^ and τ^2^). - How studies providing IPD and not providing IPD were analysed together (where applicable). - How missing data within the IPD were dealt with (where applicable). | 10-12 |
| Exploration of variation in effects | A2 | If applicable, describe any methods used to explore variation in effects by study or participant level characteristics (such as estimation of interactions between effect and covariates). State all participant-level characteristics that were analysed as potential effect modifiers, and whether these were pre-specified. | 10,11 |
| Risk of bias across studies | 15 | Specify any assessment of risk of bias relating to the accumulated body of evidence, including any pertaining to not obtaining IPD for particular studies, outcomes or other variables. | - |
| Additional analyses | 16 | Describe methods of any additional analyses, including sensitivity analyses. State which of these were pre-specified. | 11,12 |
| **Results** | | | |
| Study selection and IPD obtained | 17 | Give numbers of studies screened, assessed for eligibility, and included in the systematic review with reasons for exclusions at each stage. Indicate the number of studies and participants for which IPD were sought and for which IPD were obtained. For those studies where IPD were not available, give the numbers of studies and participants for which aggregate data were available. Report reasons for non-availability of IPD. Include a flow diagram. | 7,13 |
| Study characteristics | 18 | For each study, present information on key study and participant characteristics (such as description of interventions, numbers of participants, demographic data, unavailability of outcomes, funding source, and if applicable duration of follow-up). Provide (main) citations for each study. Where applicable, also report similar study characteristics for any studies not providing IPD. | 13 |
| IPD integrity | A3 | Report any important issues identified in checking IPD or state that there were none. | - |
| Risk of bias within studies | 19 | Present data on risk of bias assessments. If applicable, describe whether data checking led to the up-weighting or down-weighting of these assessments. Consider how any potential bias impacts on the robustness of meta-analysis conclusions. | 13 |
| Results of individual studies | 20 | For each comparison and for each main outcome (benefit or harm), for each individual study report the number of eligible participants for which data were obtained and show simple summary data for each intervention group (including, where applicable, the number of events), effect estimates and confidence intervals. These may be tabulated or included on a forest plot. | 13, Tables |
| Results of syntheses | 21 | Present summary effects for each meta-analysis undertaken, including confidence intervals and measures of statistical heterogeneity. State whether the analysis was pre-specified, and report the numbers of studies and participants and, where applicable, the number of events on which it is based. | 13,14 |
|  |  | When exploring variation in effects due to patient or study characteristics, present summary interaction estimates for each characteristic examined, including confidence intervals and measures of statistical heterogeneity. State whether the analysis was pre-specified. State whether any interaction is consistent across trials. |  |
|  |  | Provide a description of the direction and size of effect in terms meaningful to those who would put findings into practice. |  |
| Risk of bias across studies | 22 | Present results of any assessment of risk of bias relating to the accumulated body of evidence, including any pertaining to the availability and representativeness of available studies, outcomes or other variables. | - |
| Additional analyses | 23 | Give results of any additional analyses (e.g. sensitivity analyses). If applicable, this should also include any analyses that incorporate aggregate data for studies that do not have IPD. If applicable, summarise the main meta-analysis results following the inclusion or exclusion of studies for which IPD were not available. | 14 |
| **Discussion** | | | |
| Summary of evidence | 24 | Summarise the main findings, including the strength of evidence for each main outcome. | 15 |
| Strengths and limitations | 25 | Discuss any important strengths and limitations of the evidence including the benefits of access to IPD and any limitations arising from IPD that were not available. | 15-18 |
| Conclusions | 26 | Provide a general interpretation of the findings in the context of other evidence. | 19 |
| Implications | A4 | Consider relevance to key groups (such as policy makers, service providers and service users). Consider implications for future research. | 17,18 |
| **Funding** | | | |
| Funding | 27 | Describe sources of funding and other support (such as supply of IPD), and the role in the systematic review of those providing such support. | 20,21 |

# Supplementary Methods: respiratory parameters – calculations.

V_T_ was expressed in ml kg^-1^ predicted body weight [PBW], in males using the equation 50 + [0.91 × height(cm) – 152.4] and for females: 45.5 + [0.91 × height(cm) − 152.4]. Driving pressure (ΔP) was calculated using the equations: ΔP = Pplat – PEEP (for volume–controlled ventilation [VCV]) or ΔP = Pinsp – PEEP (for pressure–controlled ventilation [PCV]).^12^ Mechanical power of ventilation (MP) was calculated using the equations: MP = 0.098 * Vt * RR* (Ppeak – 0.5 * ΔP) (for VCV),^13, 14^ or MP = 0.098 * Vt * RR * (Pinsp – 0.5 * ΔP) (with PCV).^15^ The respiratory system compliance (C_RS_) was calculated by the ratio of V_T_ to the ΔP.

# Supplementary Methods: Propensity score matching

For all subjects, a propensity score was estimated with logistic regression and used to match male and female subjects with similar disease severity. Baseline characteristics implemented in the propensity score matching were chosen on clinical relevance–including age, BMI, compliance, COPD, and type of surgery. Missing data was imputed using multiple imputations if: (1) data was considered missing entirely at random and (2) not exceeding 5% of all observations. A maximum caliper of 0.02 was used. The method of nearest neighbour matching without replacement was applied in a 1:1 ratio. The balance of covariates between the two groups was assessed using LOVE plots. Three sensitivity analyses were performed. First, the primary analysis was repeated in patients with a BMI >30 kg m^-2^ and patients with a BMI <30 kg m^-2^. Second, to determine if the findings could be influenced by the level of PEEP, and finally using the ARISCAT risk score as an interaction term.

# Hierarchy of clinical importance analysis

Since the primary outcome consists of several binary pulmonary complications, we also performed an additional analysis to investigate the hierarchy of clinical importance within the individual components of both the composite outcome PPCs as well as the combination of PPCs and patient–centred outcomes, as a way to provide insights on the interpretation of outcomes and provide information for future trials design. Hence, we used the Finkelstein-Schoenfeld method in these analyses to compare hierarchical endpoints for the unmatched cohort in order of their clinical importance; 1) postoperative ventilation, severe PPCs, and mild PPCs and 2) hospital mortality, hospital length of stay, postoperative ventilation, severe PPCs, and mild PPCs. The combination of patients from the normal-high etCO_2_ arm was paired with the normal to low etCO_2_ arm in accordance with this order to calculate wins, losses and ties for individual outcomes. The win ratio (WR) compares all possible combinations of patients in the normal-high etCO_2_ and low etCO_2_ leading to 'wins', 'losses', or 'ties', based on a series of endpoints. In combinations, if a patient with normal-high etCO_2_ does better, it is called win, whereas if a patient with low etCO2 does better, it is called loss. Win ratio is defined as the number of total wins divided by total losses and a win ratio <1 indicates greater harm with low etCO_2_ group compared to normal-high etCO_2_ group (based on hierarchical endpoints).

# Supplementary Table 1: Study characteristics of the PROVHILO and PROBESE

|  | **PROVHILO** | **PROBESE** |
| --- | --- | --- |
| *General study characteristics* | | |
| Centres | 30 | 70 |
| Number of patients | 894 | 1976 |
| Inclusion Criteria | Age ≥ 18 yr Open abdominal surgery under general anaesthesia ARISCAT ≥ 26 | Age ≥ 18 yr Major surgery under general anaesthesia ARISCAT ≥ 26 Expected duration >2 h BMI ≥ 35 kg/m^2^ |
| Exclusion Criteria | Laparoscopic surgery BMI > 40 kg/m^2^ Pregnancy  Previous lung surgery  Severe COPD (use of NIV and/or oxygen therapy at home, repeated systemic corticosteroids for acute exacerbation  ARDS  Use of invasive ventilation >30 min in last 30 days  Intractable shock, persistent hemodynamic instability Severe cardiac disease Immunosuppression < 60 days  Neuromuscular disease  Enrolled in other interventional study or refusal of informed consent | Neurosurgery or cardiac surgery Need of OLV Planned reintubation after surgery Prone or lateral positioning Pregnancy Previous lung surgery Severe COPD (use of NIV and/or oxygen therapy at home, repeated systemic corticosteroids for acute exacerbation  ARDS Use of invasive ventilation >30 min in last 30 days Intractable shock, persistent hemodynamic instability Severe cardiac disease Immunosuppression < 60 days Severe pulmonary arterial hypertension  Neuromuscular disease Intracranial tumour or injury  Enrolled in other interventional study or refusal of informed consent |
| *Ventilation characteristics* | | |
| High-PEEP with recruitment manoeuvres arm | Mode: VCV Tidal volume ≤ 8 ml/kg/PBW FiO2 ≥ 0.40 to target SpO2 ≥ 92% PEEP at 12 cm H2O Recruitment manoeuvres (after endotracheal intubation, after any disconnection from the ventilator, and just before tracheal extubation) I:E 1:2 RR to etCO2 4.5–6.0 kPa (34-45 mmHg) | Mode: VCV Tidal volume ≤ 7 ml/kg/PBW FiO2 ≥ 0.40 to target SpO2 ≥ 92% PEEP at 12 cm H_2_O Recruitment manoeuvres (after endotracheal intubation, repeated every hour after any disconnection from the mechanical ventilator, and before the end of surgery) I:E 1:2 RR to etCO2 4.5–6.0 kPa (34-45 mmHg) |
| Low-PEEP arm | Mode: VCV Tidal volume ≤ 8 ml/kg PBW FiO2 > 0.40 to SpO2 ≥92% PEEP ≤ 2 cm H2O No recruitment manoeuvres I:E 1:2 RR to etCO2 4.5–6.0 kPa (34-45 mmHg) | Mode: VCV Tidal volume ≤ 7 ml/kg PBW FiO2 > 0.40 to SpO2 ≥92% PEEP at 4 cm H2O No recruitment manoeuvres I:E 1:2 RR to etCO2 4.5–6.0 kPa (34-45 mmHg) |
| *Outcomes* | | |
| Primary outcome | Incidence of PPCs | Incidence of PPCs |
| Secondary outcomes | Intraoperative complications Unexpected ICU admission Hospital-free days at day 90 Postoperative wound healing  Postoperative extrapulmonary complications Mortality | Incidence of severe PPCs  Intraoperative complications Unexpected ICU admission Hospital-free days at day 90 Postoperative wound healing  Postoperative extrapulmonary complications Mortality |
| *Methods* | | |
| Power | 80% | 80% |
| Effect size | 7.5% absolute risk reduction | 10% absolute risk reduction |
| Alpha | Two sided, 0.05 | Two sided, 0.05 |
| Interim analysis | Twice, at recruitment of 300 and 500 patients | Twice, at recruitment of 50% and 75% of total patients |
| Stopping rules | O'Brien–Fleming | Lan–DeMets alpha-spending function |
| Blinding | Double-blinded | Double-blinded |
| Statistical methods | Intention-to-treat | Modified intention-to-treat |
| *Randomisation* | | |
| Method | Permuted block randomisation with variable block sizes and stratified by centre | Permuted block randomisation with variable block sizes and stratified by centre |
| Allocation concealment | Central and web-base, available 24h/day | Central and web-base, available 24h/day |
| *Risk of Bias* |  |  |
| Low risk | Random sequence generation; allocation concealment; blinding- outcomes; incomplete outcome data, selective reporting, other source | Random sequence generation; allocation concealment; blinding- outcomes; incomplete outcome data, selective reporting, other source |
| High-risk | Blinding of participants or personnel | Blinding of participants or personnel |

ARDS, acute respiratory distress syndrome; ARISCAT, Assess Respiratory Risk in Surgical Patients in Catalonia; ARR, absolute risk reduction; Cdyn, dynamic compliance; COPD, chronic obstructive pulmonary disease; NIV, noninvasive ventilation; OLV, one-lung ventilation; ICU, intensive care unit; FiO_2_: fraction of inspired oxygen, PEEP: positive end-expiratory pressure; PBW, predicted body weight; PEPC, postoperative extrapulmonary complications; PPCs, postoperative pulmonary complications; PROBESE, protective intraoperative ventilation in obese patients; PROVHILO, protective ventilation during general anaesthesia for open abdominal surgery; VCV, volume-controlled ventilation; RR, respiratory rate.

# Supplementary Table 2: Data collected in the original studies of the PROVHILO and PROBESE

| Age |
| --- |
| Sex |
| Height and weight |
| Body mass index |
| ARISCAT score |
| Risk factors for PPCs (SpO_2_%, respiratory infection within the last month, anaemia, position of surgical incision) |
| ASA physical status classification |
| New York Heart Association classification |
| Functional status |
| Coexisting conditions (Heart failure, chronic obstructive pulmonary disease, cancer) |
| Preoperative blood tests |
| Type and duration of surgery |
| Surgical approach, incision, and priority of the surgery |
| Antibiotic prophylaxis |
| Open lung approach |
| Protocolized postoperative intervention |
| Type and duration of anesthesia |
| Day of week of start of anesthesia |
| Time of day of start of anesthesia |
| Intraoperative patient characteristics (temperature, heart rate, blood pressure, mean arterial pressure, urine output, blood loss, iv fluids, blood products) |
| Intraoperative ventilation characteristics (FiO_2_, tidal volume, PEEP, peak pressure, calculated Cdyn, respiratory rate, recruitment manoeuvre, minute ventilation, SpO_2_, etCO_2_)­– at the beginning, and hourly during the procedure |
| *Outcomes:* Intraoperative complications––arrhythmia, hypotension, hypoxemia, need for rescue manoeuvre, Unexpected ICU admission, hospital length of stay, hospital mortality |

ARISCAT: Assess Respiratory Risk in Surgical Patients in Catalonia; ASA: American society of Anesthesiologists; PPCs: postoperative pulmonary complications; Cdyn- dynamic compliance; PEEP: Positive end-expiratory pressure; etCO_2_: End-tidal carbon-dioxide; FiO2: Fraction of inspired oxygen; SpO_2_: oxygen saturation; SIRS: Systemic inflammatory response syndrome; ICU: Intensive care unit

# Supplementary Table 3: Definitions of the outcomes in the original study

|  | **PROVHILO** | **PROBESE** |
| --- | --- | --- |
| **Postoperative pulmonary complications** | | |
| Mild respiratory failure | PaO_2_ < 8 Kpa or SpO_2_ < 90% breathing at least 10 minutes of room air but responding to supplemental oxygen of 2 L/minute | PaO_2_ < 8 Kpa or SpO_2_ < 90% breathing at least 10 minutes of room air but responding to supplemental oxygen of 2 L/minute |
| Moderate respiratory failure |  | PaO_2_ < 8 Kpa or SpO_2_ < 90% despite supplemental oxygen |
| Severe respiratory failure | PaO_2_ < 8 Kpa or SpO_2_ < 90% despite supplemental oxygen or need for non-invasive or invasive mechanical ventilation | Need for non-invasive or invasive mechanical ventilation |
| ARDS | AECC criteria^a^ | Berlin criteria^b^ |
| Pulmonary infection | Need of antibiotics and at least one of the following criteria: new or changed sputum, new or changed lung opacities on chest X-ray when clinically indicated, tympanic temperature >38∙3°C, WBC count >12,000/μl in the absence of other infectious focus | Presence of a new pulmonary infiltrate and/or progression of previous pulmonary infiltrates on a chest radiograph plus at least two of the following criteria: (a) leucocytosis with > 12,000 WBC/mm^3^ or leukopenia with < 4000 WBC/mm^3^, (b) fever > 38°C or hypothermia < 36°C, and (c) increased secretions with purulent sputum, (d) antibiotic treatment |
| Pleural effusion | Chest radiography with the blunting of the costophrenic angle, displacement of the adjacent anatomical structures, and blunting of the hemidiaphragmatic silhouette in the upright position, or a hazy opacity in one of the hemithorax and preserved vascular shadows | Chest radiography showing blunting of the costophrenic angle, displacement of adjacent anatomical structures, and blunting of the hemidiaphragmatic silhouette in the upright position, or a hazy opacity in one of the hemithorax (in supine position) and preserved vascular shadows |
| Atelectasis | Chest radiography demonstrating lung opacification with shift of the mediastinum, hilum, or hemidiaphragm towards the affected area, and compensatory overinflation in the adjacent non-atelectatic lung | Chest radiography with lung opacification with shift of the mediastinum, hilum, or hemidiaphragm towards the affected area, and compensatory overinflation in the adjacent non-atelectatic lung |
| Pneumothorax | Chest radiography with air in the pleural space with no vascular bed surrounding the visceral pleura | Chest radiography with air in the pleural space with no vascular bed surrounding the visceral pleura |
| Bronchospasm | Presence of expiratory wheezing treated with bronchodilator | Presence of expiratory wheezing treated with bronchodilator |
| Aspiration Pneumonitis | Respiratory failure due to aspiration of regurgitated gastric contents | Respiratory failure due to aspiration of regurgitated gastric contents |
| Cardiopulmonary oedema | Clinical signs of congestion such as dyspnoea, oedema, rales or jugular venous distention and chest X-ray showing diffuse alveolar infiltrates and increased vascular markings | Clinical signs of congestion such as dyspnoea, oedema, rales or jugular venous distention and chest X-ray showing diffuse alveolar infiltrates and increased vascular markings |
| New pulmonary infiltrate | Monoliteral or bilateral infiltrate on the chest X-ray without other clinical signs | Monoliteral or bilateral infiltrate on the chest X-ray without other clinical signs |

| **Intraoperative complications** | | |
| --- | --- | --- |
| Hypotension | Systolic blood pressure < 90 mmHg for >3 min | During recruitment manoeuvres and defined by systolic blood pressure < 90 mmHg for >2 min |
| Hypoxemia | SpO2 <90% and any manoeuvre to rescue the SpO2 of the patient outside the proposed protocol | SpO2≤92% for >1 minute or any manoeuvre to rescue the SpO2 of the patient outside the proposed protocol |
| Need for vasoactive drugs | Any use of unplanned vasoactive drugs to rescue the blood pressure of the patient | Any use of unplanned vasoactive drugs to rescue the blood pressure of the patient |
| Arrhythmias | Any new arrhythmias needing intervention | Bradycardia (heart rate <50 beats/min or a decrease >20% if the baseline heart rate was <50 beats/min before a recruitment maneuver |
| Massive transfusion | >5 units of packed-red-blood cells during 1 h |  |
| Any surgical complication |  |  |
| ARDS: acute respiratory distress syndrome; SIRS: systemic inflammatory response syndrome; WBC: White blood cell, SpO_2_: peripheral oxygen saturation; PaO_2_: partial pressure of oxygen; GCS: Glasgow coma score; DIC: disseminated intravascular coagulation  ^a^ Bernard GR, Artigas A, Brigham KL, et al. Report of the American-European consensus conference on ARDS: definitions, mechanisms, relevant outcomes and clinical trial coordination. The Consensus Committee. Intensive Care Med 1994;20:225–32.  ^b^ Ranieri VM, Rubenfeld GD, Thompson BT, et al. Acute respiratory distress syndrome: the Berlin Definition. JAMA 2012;307:2526–33  ^c^ Bellomo R, Ronco C, Kellum JA, et al. Acute renal failure - definition, outcome measures, animal models, fluid therapy and information technology needs: the Second International Consensus Conference of the Acute Dialysis Quality Initiative (ADQI) Group. Crit Care 2004;8:R204–12  ^d^ Mehta RL, Kellum JA, Shah SV, et al. Acute kidney injury network: acute kidney injury network: report of an initiative to improve outcomes in acute kidney injury. Crit Care 2007;11:R31 | | |

| Supplementary Table 4. Ventilation characteristics (after one hour) | | | | | | | | | |
| --- | --- | --- | --- | --- | --- | --- | --- | --- | --- |
| **Characteristics** | **Overall** | | | **Low PEEP (normal)** | | | **High PEEP (intervention)** | | |
|  | **low etCO_2_**  **N = 821** | **Normal/high etCO_2_**  **N = 1972** | ***P value*** | **low etCO_2_**  **N = 821** | **Normal/high etCO_2_**  **N = 1972** | ***P value*** | **low etCO_2_**  **N = 821** | **Normal/high etCO_2_**  **N = 1972** | ***P value*** |
| Tidal volume, ml/kg PBW | 7.8 [7.1 to 8.1] | 7.1 [7.0 to 7.5] | <0.001 | 7.8 [7.1 to 8.1] | 7.1 [7.0 to 7.5] | <0.001 | 7.8 [7.1 to 8.1] | 7.1 [7.0 to 7.5] | <0.001 |
| Respiratory rate, breaths/min | 12 [10 to 14] | 15 [12 to 18] | <0.001 | 12 [10 to 14] | 16 [12 to 18] | <0.001 | 12 [10 to 14] | 15 [12 to 18] | <0.001 |
| <10 | 84 (10%) | 98 (6%) | <0.001 | 44 (10%) | 45 (5%) | <0.001 | 40 (11%) | 53 (5%) | <0.001 |
| 10–15 | 623 (76%) | 886 (45%) |  | 342 (77%) | 424 (44%) |  | 281 (75%) | 461 (47%) |  |
| >15 | 112 (14%) | 967 (49%) |  | 59 (13%) | 500 (51%) |  | 53 (14%) | 467 (48%) |  |
| FiO_2_, % | 43 [40 to 50] | 42 [40 to 50] | 0.95 | 44 [40 to 50] | 44 [40 to 50] | 0.01 | 42 [40 to 50] | 40 [40 to 50] | 0.01 |
| etCO_2_, kPa | 4.4 [4.1 to 4.7] | 5.3 [5.1 to 5.9] | <0.001 | 4.4 [4.1 to 4.7] | 5.3 [4.9 to 5.7] | <0.001 | 4.5 [4.3 to 4.7] | 5.5 [5.1 to 5.9] | <0.001 |
| Respiratory system compliance^‡^, ml/cmH_2_O | 36 [27 to 49] | 30 [23 to 40] | <0.001 | 31 [22 to 44] | 25 [18 to 35] | <0.001 | 46 [37 to 59] | 40 [33 to 50] | <0.001 |
| PEEP, cmH_2_O | 4 [2 to 12] | 11 [4 to 12] | <0⋅001 | 2 [1 to 4] | 4 [4 to 4] | <0⋅001 | 12 [12 to 12] | 12 [12 to 12] | 0.37 |
| Pplat*, cmH_2_O | 22 [19 to 25] | 23 [20 to 25] | 0.12 | 21 [18 to 24] | 22 [19 to 25] | 0.12 | 23 [20 to 27] | 24 [21 to 26] | 0.92 |
| Ppeak, cmH_2_O | 22 [18 to 26] | 26 [23 to 30] | <0.001 | 19 [15 to 24] | 25 [21 to 29] | <0.001 | 24 [22 to 27] | 27 [24 to 30] | <0.001 |
| Driving pressure, cmH_2_O | 14 [10 to 18] | 15 [11 to 19] | 0.42 | 17 [14 to 20] | 18 [15 to 21] | 0.02 | 12 [9 to 14] | 11 [8 to 15] | 0.57 |
| Power, J/min | 11 [9 to 14] | 13 [10 to 16] | <0.001 | 10 [8 to 12] | 11 [9 to 14] | <0.001 | 13 [10 to 16] | 14 [12 to 18] | 0.25 |
| Minute volume (normalised to body weight), ml/kg/min | 69 [57 to 83] | 60 [51 to 69] | <0.001 | 69 [57 to 82] | 60 [51 to 69] | <0.001 | 69 [56 to 84] | 59 [50 to 68] | <0.001 |
| <60 | 239 (31%) | 857 (50%) | <0.001 | 130 (31%) | 402 (48%) | <0.01 | 109 (31%) | 455 (53%) | <0.001 |
| 60–80 | 298 (39%) | 686 (41%) |  | 167 (40%) | 355 (42%) |  | 131 (38%) | 331 (38%) |  |
| 80–100 | 170 (22%) | 134 (8%) |  | 94 (23%) | 73 (9%) |  | 76 (22%) | 61 (7%) |  |
| >100 | 55 (8%) | 26 (1%) |  | 24 (6%) | 11 (1%) |  | 31 (9%) | 15 (2%) |  |
| Abbreviations: PBW: Predicted body weight, FiO_2:_ Fraction of inspired oxygen score, etCO_2_: End–tidal carbon dioxide, PEEP: Positive end–expiratory pressure, Pplat: Plateau pressure, Ppeak: Peak pressure, DP: Driving pressure; *Pplat was available only for the PROBESE study. ^‡^Respiratory system compliance = tidal volume/driving pressure | | | | | | | | | |
| Supplementary Table 5. Outcome in low and high PEEP groups | | | | | | | | | |
| **Characteristics** | **Overall** | | | **Low PEEP (normal)** | | | **High PEEP (intervention)** | | |
|  | **low etCO_2_**  **N = 821** | **Normal/high etCO_2_**  **N = 1972** | ***P value*** | **low etCO_2_**  **N = 446** | **Normal/high etCO_2_**  **N = 969** | ***P value*** | **low etCO_2_**  **N = 374** | **Normal/high etCO_2_**  **N = 982** | ***P value*** |
| PPCs, n (%) | 278 (34%) | 462 (23%) | <0.001 | 148 (33%) | 238 (25%) | <0.001 | 130 (35%) | 217 (22%) | <0.001 |
| Bronchospasm | 28 (3.4%) | 26 (1%) | <0.001 | 16 (4%) | 11 (1%) | 0.002 | 12 (3%) | 14 (1%) | 0.03 |
| Pulmonary infections | 71 (8.6%) | 66 (3%) | <0.001 | 39 (9%) | 35 (4%) | <0.001 | 32 (9%) | 31 (3%) | <0.001 |
| Aspiration | 4 (0.5%) | 3 (0.2%) | 0.11 | 3 (1%) | 1 (0%) | 0.06 | 1 (0%) | 2 (0%) | 0.82 |
| Atelectasis | 56 (6.8%) | 114 (5.8%) | 0.29 | 32 (7%) | 61 (6%) | 0.52 | 24 (6%) | 52 (5%) | 0.42 |
| ARDS | 5 (0.6%) | 6 (0.3%) | 0.24 | 4 (1%) | 2 (0%) | 0.06 | 1 (0%) | 4 (0%) | 0.70 |
| Pleural effusion | 106 (13%) | 102 (5%) | <0.001 | 61 (14%) | 37 (4%) | <0.001 | 45 (12%) | 62 (6%) | <0.001 |
| Mild respiratory failure | 175 (21%) | 297 (15%) | <0.001 | 88 (20%) | 159 (16%) | 0.11 | 87 (23%) | 135 (14%) | <0.001 |
| Severe respiratory failure | 64 (8%) | 122 (6%) | 0.11 | 40 (9%) | 62 (6%) | 0.08 | 24 (6%) | 59 (6%) | 0.78 |
| Intraoperative complications, n (%) | 539 (66%) | 1185 (60%) | 0.006 | 277 (62%) | 564 (58%) | <0.001 | 261 (70%) | 611 (62%) | 0.003 |
| Arrhythmia | 61 (7%) | 130 (7%) | 0.42 | 28 (6%) | 47 (5%) | 0.27 | 33 (9%) | 82 (8%) | 0.78 |
| Hypotension | 314 (38%) | 528 (27%) | <0.001 | 149 (33%) | 188 (19%) | <0.001 | 164 (45%) | 333 (34%) | <0.001 |
| Hypoxemia | 24 (3%) | 158 (8%) | 0.19 | 16 (4%) | 115 (12%) | 0.08 | 8 (2%) | 40 (4%) | 0.89 |
| Need for rescue manoeuvre | 54 (7%) | 217 (11%) | <0.001 | 43 (10%) | 158 (16%) | <0.001 | 11 (3%) | 58 (6%) | 0.03 |
| Unexpected ICU admission, n (%) | 163 (20%) | 110 (6%) | <0.001 | 86 (19%) | 47 (5%) | <0.001 | 76 (20%) | 62 (6%) | <0.001 |
| Hospital length of stay, n (%) | 8 [5 to 13] | 4 [3 to 7] | <0.001 | 8 [5 to 13] | 4 [3 to 6] | <0.001 | 8 [4 to 13] | 4 [3 to 7] | <0.001 |
| Hospital mortality, n (%) | 12 (2%) | 16 (1%) | 0.11 | 5 (1%) | 4 (0%) | 0.12 | 7 (2%) | 12 (1%) | 0.36 |
| Abbreviations: PBW: Predicted body weight, FiO_2:_ Fraction of inspired oxygen score, etCO_2_: End–tidal carbon dioxide, PEEP: Positive end–expiratory pressure, Pplat: Plateau pressure, Ppeak: Peak pressure, DP: Driving pressure; *Pplat was available only for the PROBESE study. ^‡^Respiratory system compliance = tidal volume/driving pressure | | | | | | | | | |

# Supplementary Table 6. Outcome in open abdomen and laparoscopic surgical approach

|  | | | | | | | | | |
| --- | --- | --- | --- | --- | --- | --- | --- | --- | --- |
| **Characteristics** | **Overall** | | | **Laparoscopic** | | | **Open abdomen** | | |
|  | **low etCO_2_**  **N = 821** | **Normal/high etCO_2_**  **N = 1972** | ***P value*** | **low etCO_2_**  **N = 157** | **Normal/high etCO_2_**  **N = 1280** | ***P value*** | **low etCO_2_**  **N = 621** | **Normal/high etCO_2_**  **N = 556** | ***P value*** |
| PPCs, n (%) | 278 (34%) | 462 (23%) | <0.001 | 31 (20%) | 252 (20%) | 0.99 | 236 (38%) | 181 (33%) | 0.05 |

# Supplementary Figure 1: Consort diagram displaying the included patients in the study

**
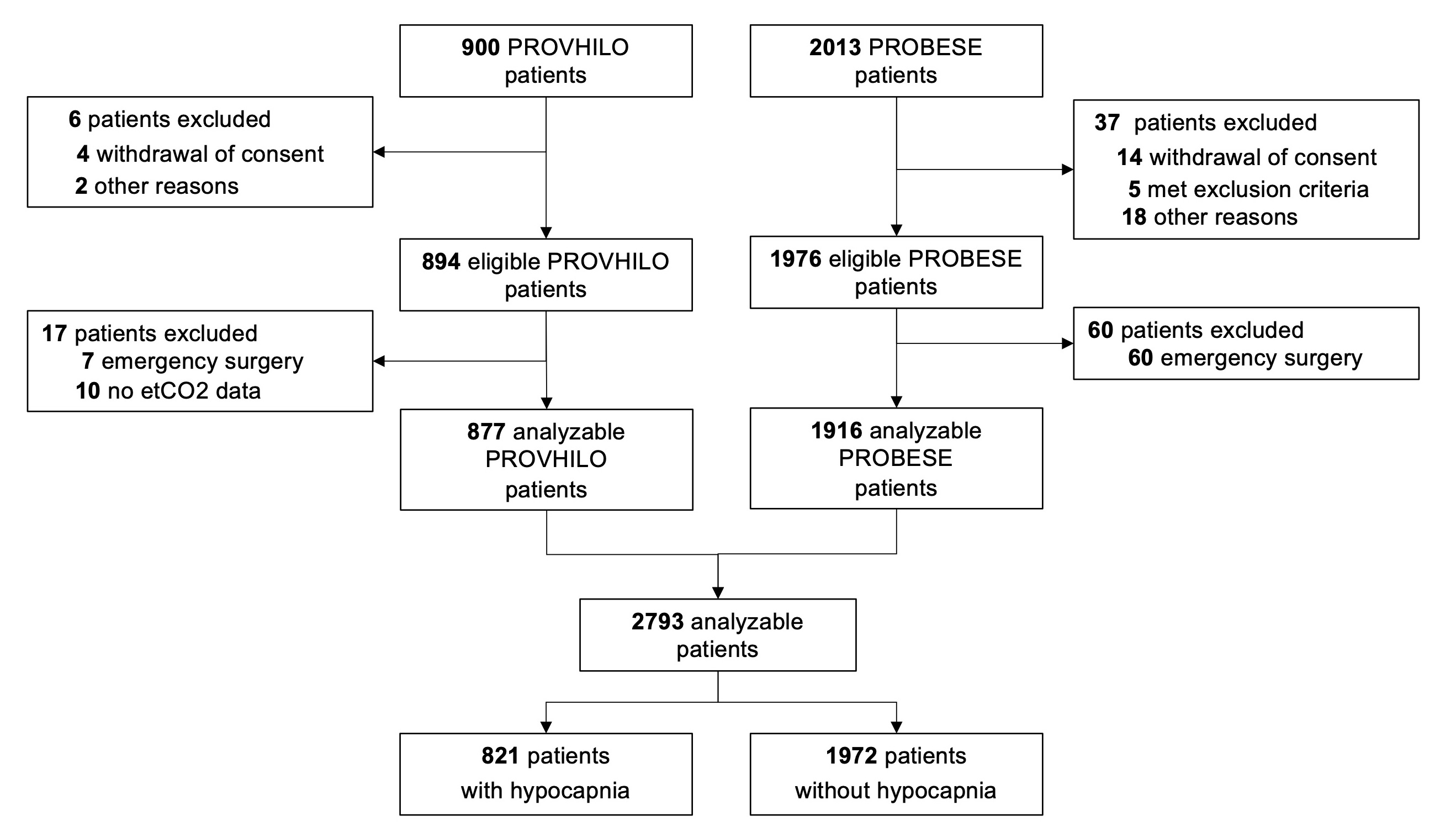
**

PROVHILO: Low Positive End–expiratory Pressure During General Anaesthesia for Open Abdominal Surgery study, PROBESE: PRotective Ventilation with Higher versus Lower PEEP during General Anesthesia for Surgery in OBESE Patients, etCO_2_**:** end-tidal carbon dioxide

# Supplementary Figure 2: Mean etCO_2_ plotted against the log odds (for PPCs)

**
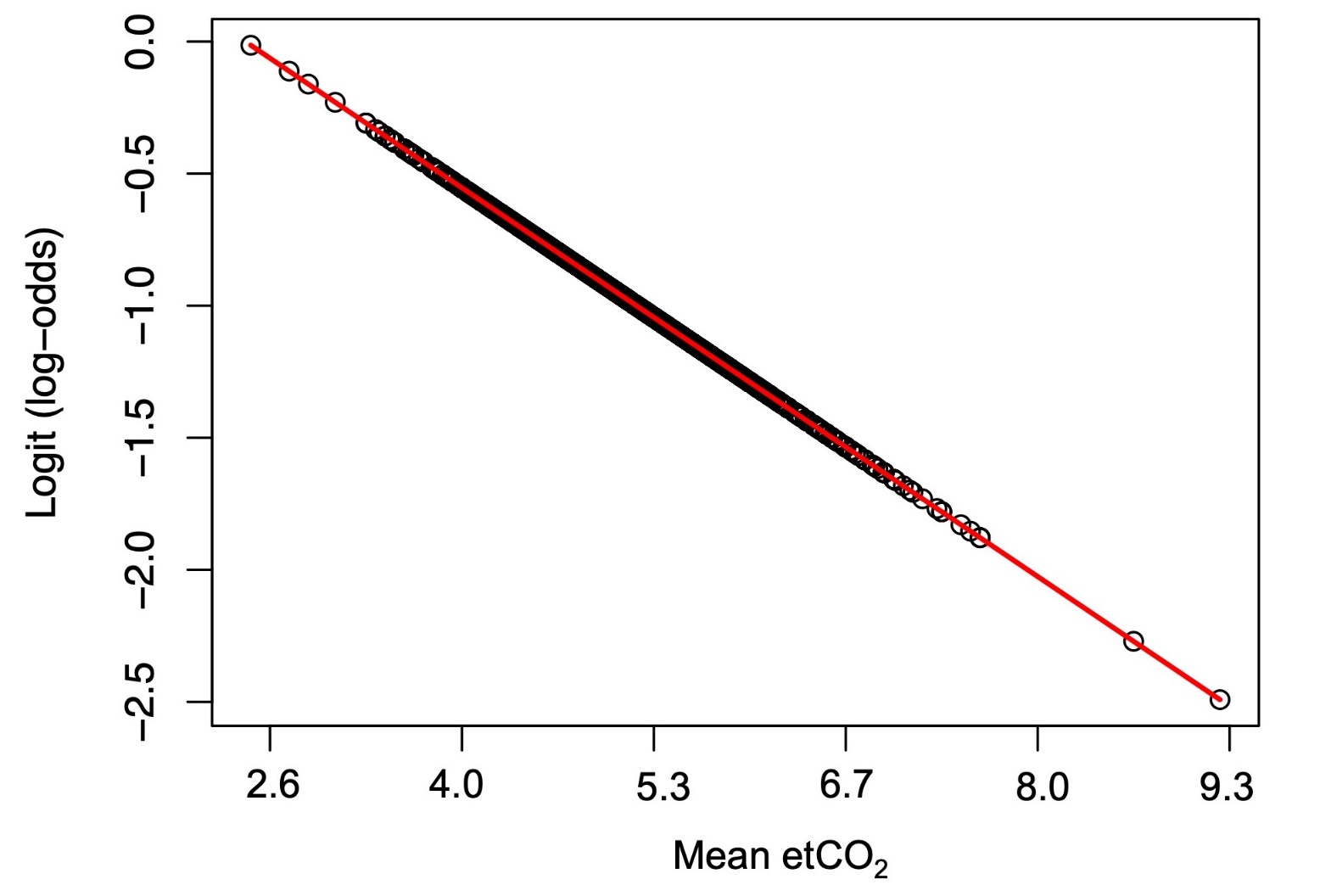
**

etCO_2_= end–tidal carbon dioxide, PPCs: postoperative complications

# Supplementary Figure 3: Love plot for assessing balance of covariates


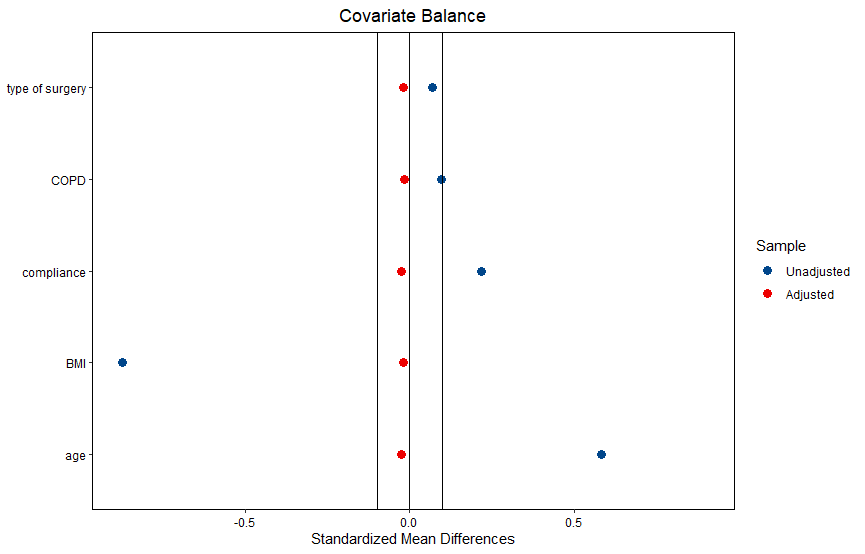


BMI: Body mass index, COPD: chronic obstructive pulmonary disease

15 Supplementary Figure 4: Distribution of Wins, Ties and Losses on pulmonary endpoints among normal to high etCO2 and low etCO2 patients
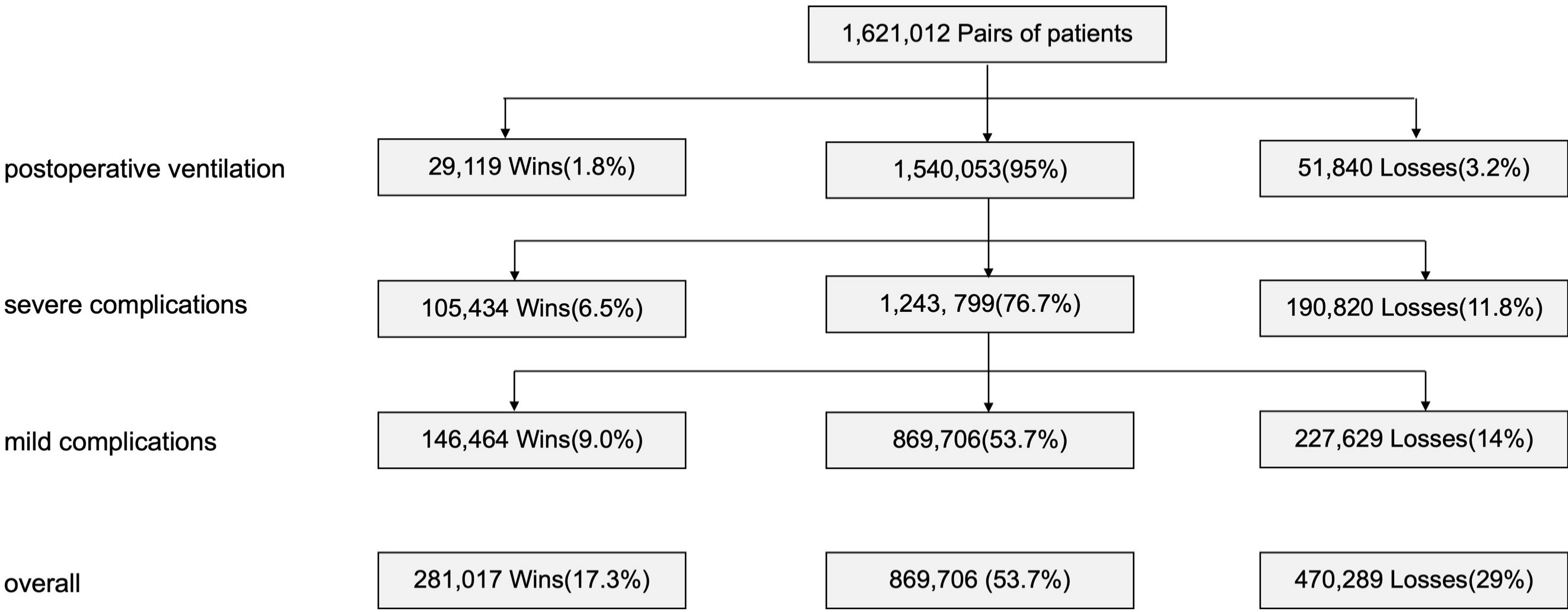


16 Supplementary Figure 5: Distribution of Wins, Ties and Losses on overall hierarchical endpoints among normal to high etCO2 and low etCO2 patients
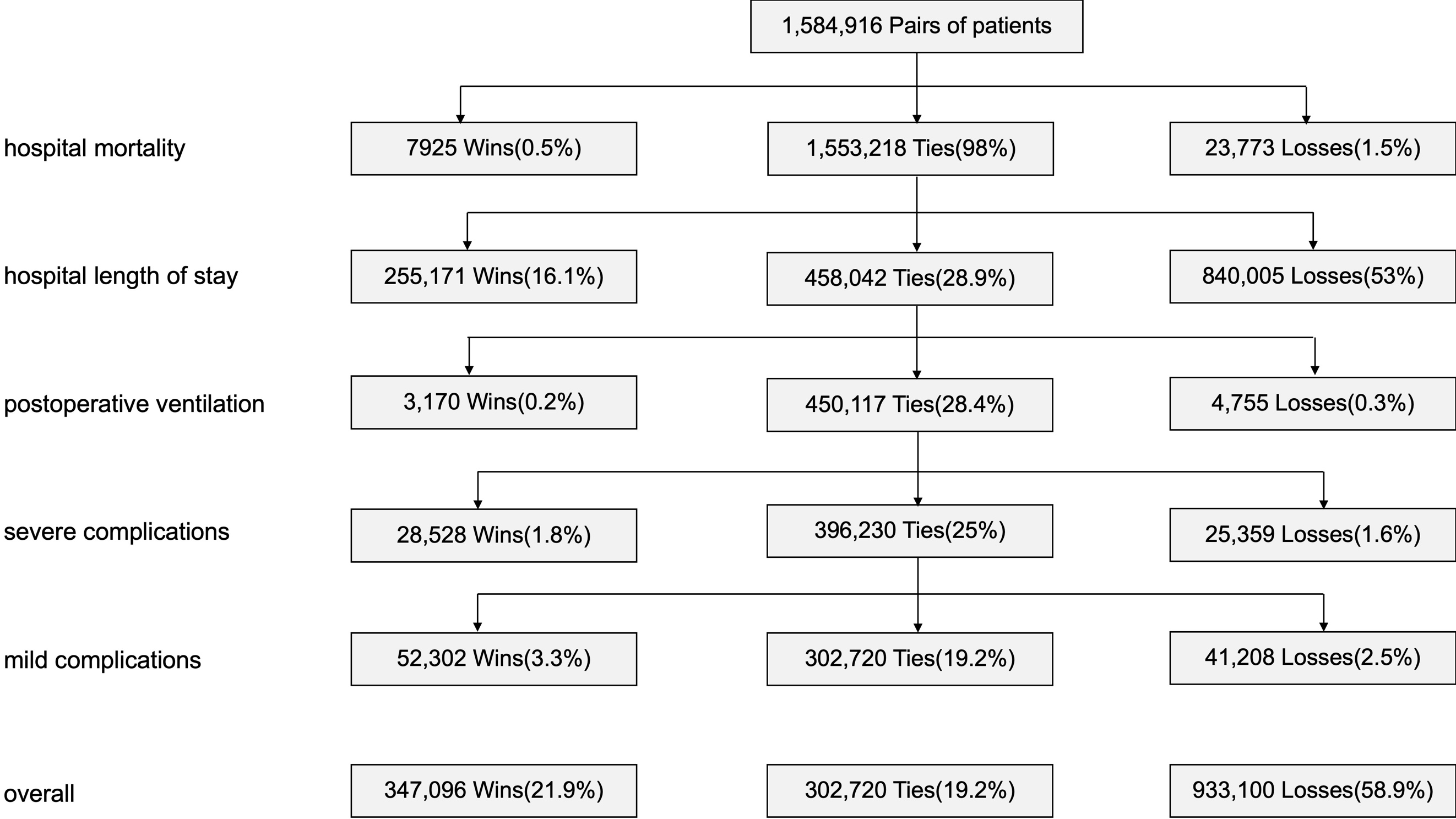

Supplement: Multimedia component 1 [file mmc1.docx]
